# Phosphatidylcholine levels regulate hyphal elongation and differentiation in the filamentous fungus *Aspergillus oryzae*

**DOI:** 10.1038/s41598-024-62580-4

**Published:** 2024-05-22

**Authors:** Tetsuki Suzawa, Ryo Iwama, Ryouichi Fukuda, Hiroyuki Horiuchi

**Affiliations:** 1 Department of Biotechnology, The University of Tokyo, Yayoi 1-1-1, Bunkyo-Ku, Tokyo, 113-8657 Japan; 2 Collaborative Research Institute for Innovative Microbiology, The University of Tokyo, Yayoi 1-1-1, Bunkyo-Ku, Tokyo, 113-8657 Japan

**Keywords:** Fungi, Fungal biology, Fungal genetics

## Abstract

Filamentous fungi are eukaryotic microorganisms that differentiate into diverse cellular forms. Recent research demonstrated that phospholipid homeostasis is crucial for the morphogenesis of filamentous fungi. However, phospholipids involved in the morphological regulation are yet to be systematically analyzed. In this study, we artificially controlled the amount of phosphatidylcholine (PC), a primary membrane lipid in many eukaryotes, in a filamentous fungus *Aspergillus oryzae*, by deleting the genes involved in PC synthesis or by repressing their expression. Under the condition where only a small amount of PC was synthesized, *A. oryzae* hardly formed aerial hyphae, the basic structures for asexual development. In contrast, hyphae were formed on the surface or in the interior of agar media (we collectively called substrate hyphae) under the same conditions. Furthermore, we demonstrated that supplying sufficient choline to the media led to the formation of aerial hyphae from the substrate hyphae. We suggested that acyl chains in PC were shorter in the substrate hyphae than in the aerial hyphae by utilizing the strain in which intracellular PC levels were controlled. Our findings suggested that the PC levels regulate hyphal elongation and differentiation processes in *A. oryzae* and that phospholipid composition varied depending on the hyphal types.

## Introduction

Biological membranes, a major constituent of cells, consist primarily of phospholipid bilayers, and the membranes compartmentalize the cells and organelles by forming external and internal boundaries^1,2^. Also, they play pivotal roles in various cellular processes driven by the formation of membrane domains, and the interactions between proteins and lipids^1^. Phospholipids are composed of hydrophilic head groups and hydrophobic tails, which are structurally diverse. Based on their head groups, phospholipids are classified into various types, such as phosphatidylcholine (PC), phosphatidylethanolamine (PE), phosphatidylserine (PS), and phosphatidylinositol (PI). The phospholipid synthesis pathways have been extensively studied in the yeast *Saccharomyces cerevisiae*. The phospholipids are synthesized via the cytidine diphosphate-diacylglycerol (CDP-DAG) and the Kennedy pathways (Fig. S1)^3–5^. In the CDP-DAG pathway, PI is synthesized by transferring inositol to the CDP-DAG by Pis1, and PS is synthesized by displacing CMP from CDP-DAG with serine by Pss1 (also known as Cho1). PE is subsequently synthesized from PS via decarboxylation by Psd1 or Psd2. After that, PE is methylated by Pem1 (also known as Cho2) and converted to phosphatidyl-*N*-monomethylethanolamine (PMME), and PMME is sequentially methylated to produce phosphatidyl-*N*-dimethylethanolamine (PDME) and PC by Pem2 (also known as Opi3). In this article, we refer to the process synthesizing PC from PE as “the *N*-methylation pathway”. PE and PC are also synthesized from ethanolamine (Etn) and choline (Cho) via the Kennedy pathway, respectively. Etn and Cho are sequentially converted to phosphoethanolamine (P-Etn) and phosphocholine (P-Cho) by Eki1 and Cki1, cytidine diphosphate ethanolamine (CDP-Etn) and cytidine diphosphate choline (CDP-Cho) by Ect1 and Pct1 (also known as Cct1), and PE and PC by Ept1 and Cpt1, respectively.

Filamentous fungi are eukaryotic microorganisms and grow in a filamentous form called hyphae. The hyphae elongate their tips by establishing and maintaining hyphal polarities^6^. Some filamentous fungi are widely used to produce various enzymes, organic acids, and fermented foods, while others infect plants and animals^7–9^. Their morphological differentiation is a critical factor in determining their beneficial or detrimental properties of filamentous fungi. Therefore, it is essential to clarify the mechanisms of their morphogenesis. The phospholipid synthesis pathways have been studied in several species of filamentous fungi belonging to Ascomycota. In *Aspergillus nidulans*, the *PEM2* ortholog *choC* is indispensable for normal growth^10^. A *PSS1* ortholog is proposed to be crucial for its growth^11^. We previously reported that the deletion mutant of a *PSD2* ortholog *psdB* showed reduced intracellular PE content and formed hyphae and conidiophores with abnormal morphologies^12^. In *Pestalotiopsis microspora*, deletion of *choC* or *PEM1* ortholog *choA* resulted in defects in vegetative growth and cell wall integrity^13^. In *Aspergillus niger*, *PEM1* and *PEM2* orthologs were characterized^14^. In *Fusarium graminearum*, the functions of ten orthologs of the genes involved in the phospholipid synthesis pathways in *S. cerevisiae* were analyzed, and it was indicated that the biosynthesis of PE and PC is critical for fungal vegetative growth and virulence in plants^15^. Herein, the strains defective in phospholipid synthesis often exhibited altered morphologies. Therefore, regulating phospholipid synthesis is suggested to be critical for hyphal morphogenesis in filamentous fungi. However, the physiological significance of phospholipid composition in the biological membranes of filamentous fungi remains to be determined.

During the asexual life cycle of filamentous fungi, the asexual spores in Ascomycota, conidia, undergo isotropic swelling, followed by establishing polarity^6^. Subsequently, germ tubes are extended, and hyphae are formed. Finally, mycelia are formed by hyphal tip elongation and branching^6^. In the case of mycelia grown on a solid medium, hyphae extend their tips on the surface and into the interior of the solid medium^16^. In this study, they are referred to as surface and penetrative hyphae, respectively, and collectively termed substrate hyphae. After a certain period since substrate hyphal formation, the hyphae begin to extend into the air, resulting in aerial hyphal formation^17^. Numerous conidiophores that produce conidia are formed on the footcells in the substrate hyphae^16,17^. Our previous study demonstrated a significant decrease in PC, and a marked increase in PE and highly unsaturated phospholipids during germination in *A. nidulans* and *A. oryzae*^18^. Additionally, we showed that PC was enriched in the central region of the colony. Another study reported that the deletion of *choC* retarded hyphal growth and formed swollen hyphal tips in *A. nidulans*^10^. These findings suggested that the levels of PC and PE play significant roles in the hyphal morphogenesis of filamentous fungi.

In this study, we examined the effects of varying intracellular PC levels on *A. oryzae*, an industrially important filamentous fungus. Our findings revealed that the absence of PC synthesis leads to severe growth defects, and the intracellular PC levels regulate the hyphal elongation and differentiation processes in *A. oryzae*. Further investigation suggested that the profiles of the acyl chain constituents of the phospholipids were altered depending on the type of hyphae.

## Results

### PC, PI, and PS increased as the differentiation process progressed in *A. oryzae*

In our previous study, we observed changes in lipid composition during germination of *A. oryzae* in a minimal medium, Czapek-Dox (CD) liquid medium^18^. However, *A. oryzae* does not differentiate aerial hyphae and conidia in the liquid medium. Thus, to investigate the changes of lipid composition during these differentiation processes, we cultivated *A. oryzae* on the CD agar medium.

**Figure 1:**
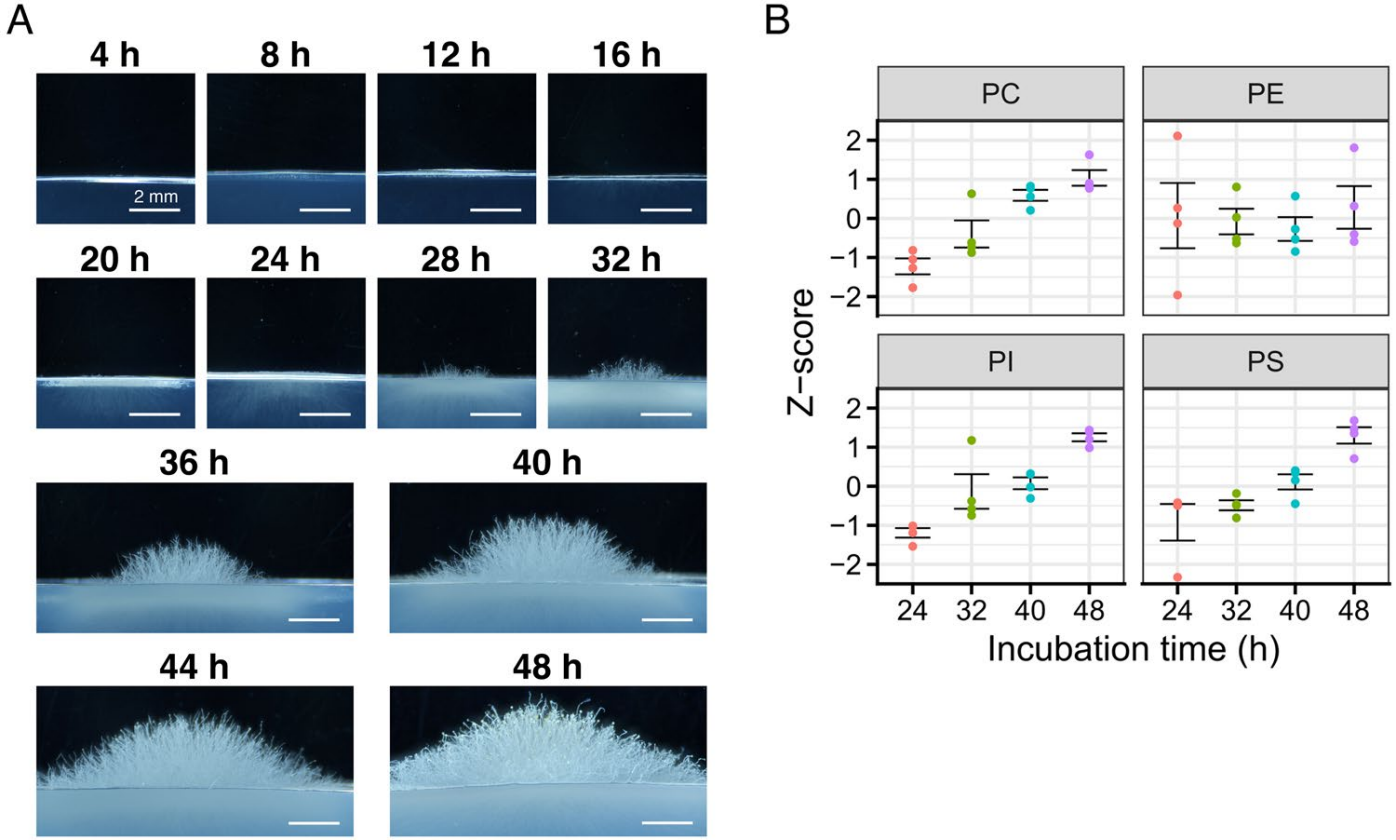
Changes in the phospholipid levels during the formation of aerial hyphae and conidiophores. (**A**) The growth of *A. oryzae* on the CD agar medium. The conidia of the wild-type strain were inoculated on the CD agar medium and incubated at 30 °C. The aerial hyphae and substrate hyphae were observed. (**B**) Changes in the phospholipid levels. Lipids were extracted from the harvested mycelia and subjected to the targeted lipidome analysis. Z-scores were calculated within each head group by utilizing the peak areas associated with each group (see “Experimental procedures” for details). The dots and error bars indicate individual data (*n* = 4), and standard errors, respectively.

The wild-type strain Δku5-2 was inoculated on the CD agar medium, and colony formation was monitored in detail (Fig. 1A). From 16 to 24 h after inoculation, only substrate hyphae grew, and no aerial hyphae were observed. Aerial hyphae emerged at 28 h after inoculation, and conidiophore formation was evident by 48 h after inoculation. To assess lipid composition changes during aerial hyphal formation, we quantified the amounts of PC, PE, PI, and PS, the major phospholipids in eukaryotes, at 24, 32, 40, and 48 h after inoculation (Fig. 1B). The amounts of PC, PI, and PS increased as the differentiation process progressed while those of PE did not change significantly.

### Defects in PC synthesis resulted in a severe growth retardation

For regulating PC levels in *A. oryzae*, we inhibited PC synthesis via the *N*-methylation pathway and bypassed PC supply via the Kennedy pathway. Although genes involved in PC synthesis in the *N*-methylation pathway have not yet been reported in *A. oryzae*, we identified genes encoding homologous proteins of Pem1 (SGDID: S000003389) and Pem2 (SGDID: S000003834) in *Saccharomyces* Genome Database (SGD, https://www.yeastgenome.org): XP_001727259.1 (BLASTP E-value: 2e-129) and XP_001818525.1 (BLASTP E-value: 4e-76), respectively, in the *A. oryzae* RIB40 genome database. These protein-encoding genes are *AO090012000204* and *AO090005001620*, and are designated as *pemA* and *pemB*, respectively. Using 5′-RACE analysis, we identified transcription initiation sites of *pemA* and *pemB* (Fig. S2A) and confirmed that *pemA* and *pemB* encode proteins comprising 971 and 202 amino acid residues, respectively. PemA is predicted to possess two phospholipid methyltransferase and ten transmembrane domains. Those features are conserved in *S. cerevisiae* Pem1. In contrast, predicted numbers of transmembrane domains differed between PemB, having five, and *S. cerevisiae* Pem2, having three, while both have one phospholipid methyltransferase domain (Fig. S2B).

**Figure 2:**
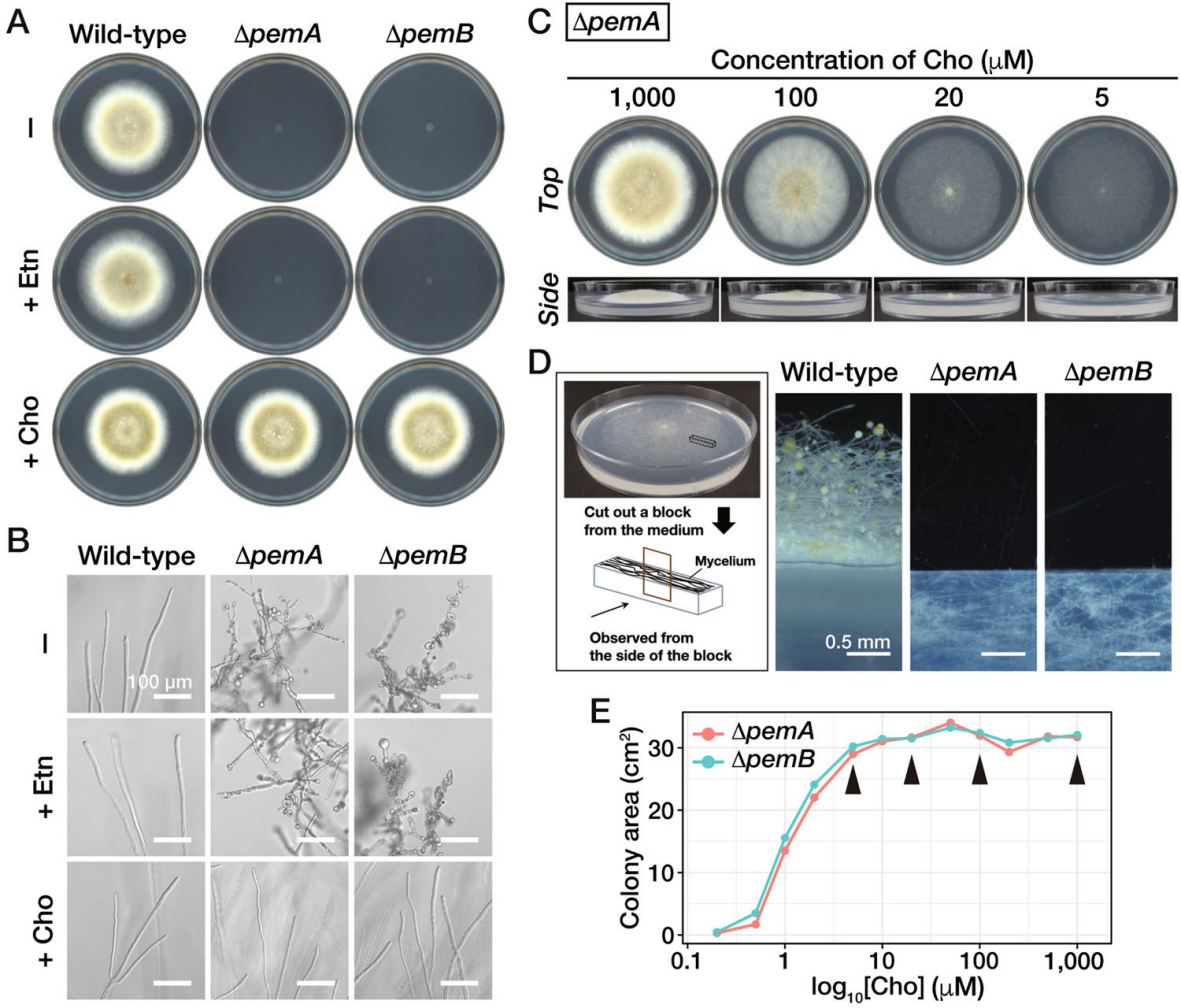
Morphologies of colonies and hyphal tips of the deletion mutant of *pemA* or *pemB*. (**A**) The conidia of the wild-type, Δ*pemA,* or Δ*pemB* strain were inoculated on a CD medium (-) or a CD medium containing 1 mM Etn (+ Etn) or 1 mM Cho (+ Cho) and incubated at 30 °C for 96 h. (**B**) The hyphae at the edge of the colonies in (**A**) were observed. (**C**) The conidia of the Δ*pemA* strain were inoculated on a CD medium containing 1 mM Etn and Cho at different concentrations: 5, 20, 100, or 1000 μM. Then, the conidia were incubated at 30 °C for 96 h. *Top* and *side* views were shown on each concentration of Cho. (**D**) The wild-type, Δ*pemA*, or Δ*pemB* strain was grown on a CD medium containing 1 mM Etn and 5 μM Cho, and their aerial hyphae and substrate hyphae were observed. The way that aerial and substrate hyphae were observed is shown in the *box*. (**E**) Colony areas were quantified at each concentration of Cho supplementation shown in Fig. S5. The conditions used in (**C**) are indicated by *arrowheads*.

We constructed deletion mutants of *pemA* and *pemB* (named the Δ*pemA* and Δ*pemB* strains, respectively) and cultivated them on the CD medium. These strains exhibited significant growth defects and produced numerous balloon-like structures near the hyphal tips (Fig. 2A and B). When we supplemented Cho in the medium, both strains regained their growth and hyphal morphology similar to the wild-type strain (Fig. 2A and B), while supplementation of Etn did not revert these phenotypes (Fig. 2A and B). These results suggested that Cho is necessary for hyphal elongation in the absence of PC synthesis via the *N*-methylation pathway and that PC can be supplied via an alternative route using Cho. There are proteins closely resembling Cki1, Pct1, and Cpt1 in the *A. oryzae* database strongly suggesting that the Kennedy pathway supplied PC in *A. oryzae* (Table S1). The Δ*pemA* and Δ*pemB* strains both exhibited similar dry cell weight (DCW) compared to the wild-type strain when cultured in the CD liquid medium containing Cho. However, the growth of these deletion mutants was severely retarded in the CD liquid medium lacking Cho (Fig. S3).

Next, we investigated the expression levels of genes involved in PC synthesis of the wild-type strain using RT-qPCR. Although genes involved in the Kennedy pathway have not yet been reported in *A. oryzae*, we identified genes encoding homologous proteins of Eki1, Ect1, Ept1, Cki1, Pct1, and Cpt1 in SGD, and designated these genes as follows; *ekiA*, *ectA*, *eptA*, *ckiA*, *cctA*, and *cptA* (Table S1). Relative to the transcript levels at 24 h, those of genes involved in the Kennedy pathway, except for *ckiA*, increased, particularly at 40 and 48 h (Fig. S4), while those of *pemA* and *pemB* decreased. These findings raise the possibility that the Kennedy pathway may be more active than the *N*-methylation pathway in the PC synthesis during aerial hyphal formation.

### PC levels regulate the formation of substrate and aerial hyphae

Subsequently, we added Cho to the CD medium at different concentrations to control PC supply in the Δ*pemA* and Δ*pemB* strains (Figs. 2C and S5). Ranging from 1000 to 10 μM, the density of aerial hyphae gradually decreased, and the height of aerial hyphae became shorter. In the presence of 5 μM supplementation, aerial hyphae were hardly formed (Fig. 2D). In contrast, the extension rate of substrate hyphae was almost the same as that of the wild-type strain under the same conditions (Fig. 2C). There was little difference in colony area between 1000 and 5 μM supplementations (Fig. 2E). Ranging from 5 to 0.5 μM, the area of substrate hyphae gradually decreased (Figs. 2E and S5). Finally, 0.2 μM Cho hardly recovered their growth defects on the CD medium (Fig. S5). These results suggested that the intracellular PC levels affected the hyphal elongation and differentiation process in *A. oryzae*. No morphological changes of the substrate hyphae were observed in either the Δ*pemA* nor Δ*pemB* strains compared to the wild-type strain when the 5–50 μM Cho was added to the medium (Fig. S6A). On the other hand, the density of substrate hyphae near the agar surface increased and aerial hyphae differentiation occurred as the concentration of Cho increased (Fig. S6B).

**Figure 3:**
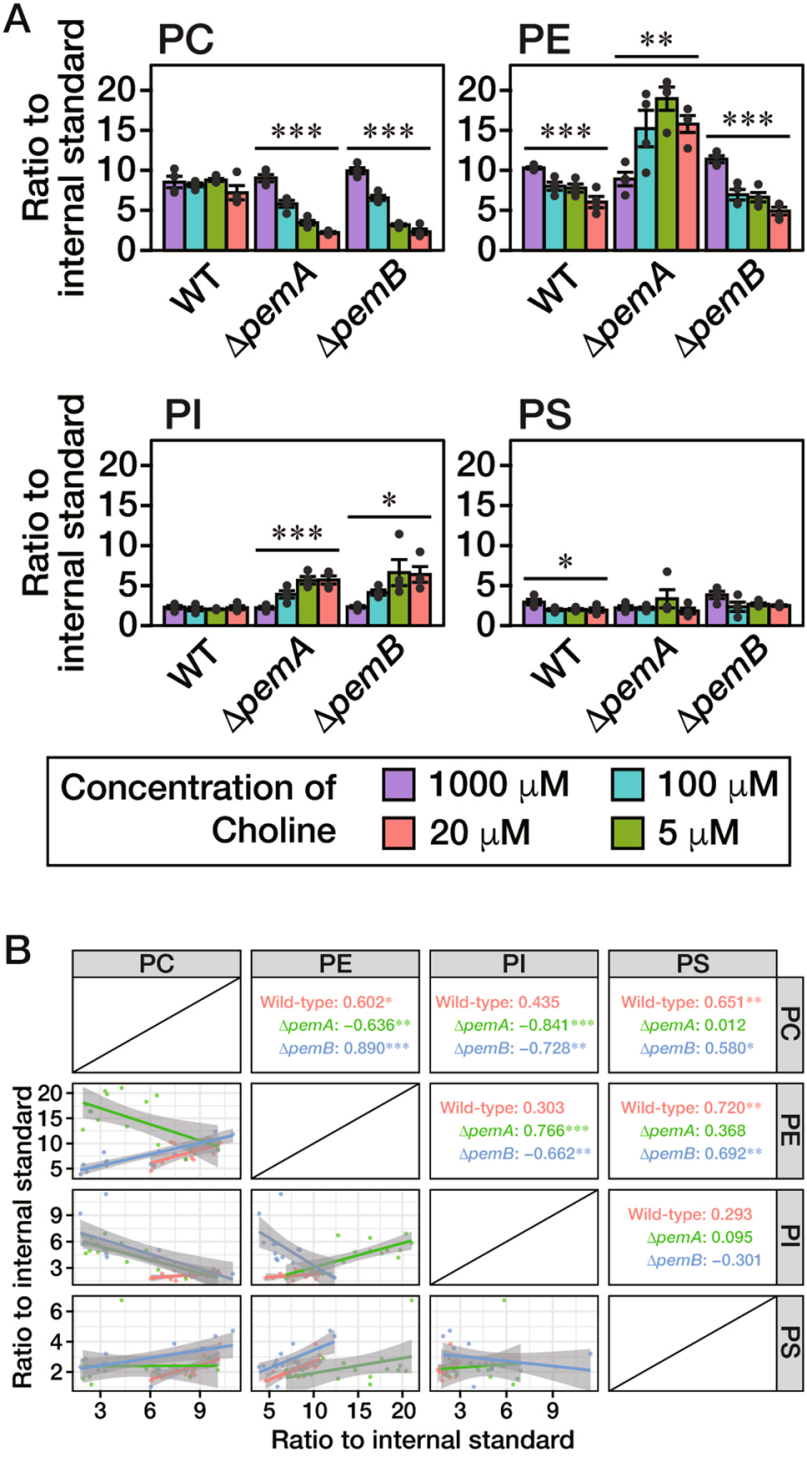
Phospholipid level of each head group at various concentrations of Cho. (**A**) The conidia of the wild-type, Δ*pemA*, or Δ*pemB* strain were inoculated on a CD medium containing 1 mM Etn and 5, 20, 100, or 1000 μM Cho covered with a cellophane sheet and incubated at 30 °C for 36 h. Phospholipids were extracted from the harvested mycelia and subjected to lipidome analysis. The mean values are depicted as bars (*n* = 4), while dots indicate individual data points. The error bars denote the standard error. Statistically significant differences are indicated by asterisks (***P < 0.001, **P < 0.01, *P < 0.05; one-way analysis of variance (one-way ANOVA)). (**B**) For each strain, the correlations between the amounts of phospholipids were examined. WT; *red*, Δ*pemA*; *green*, Δ*pemB*; *blue*. The values shown indicate the correlation coefficient for each combination. Statistically significant differences are indicated by asterisks (***P < 0.001, **P < 0.01, *P < 0.05; test of no correlation).

**Figure 4:**
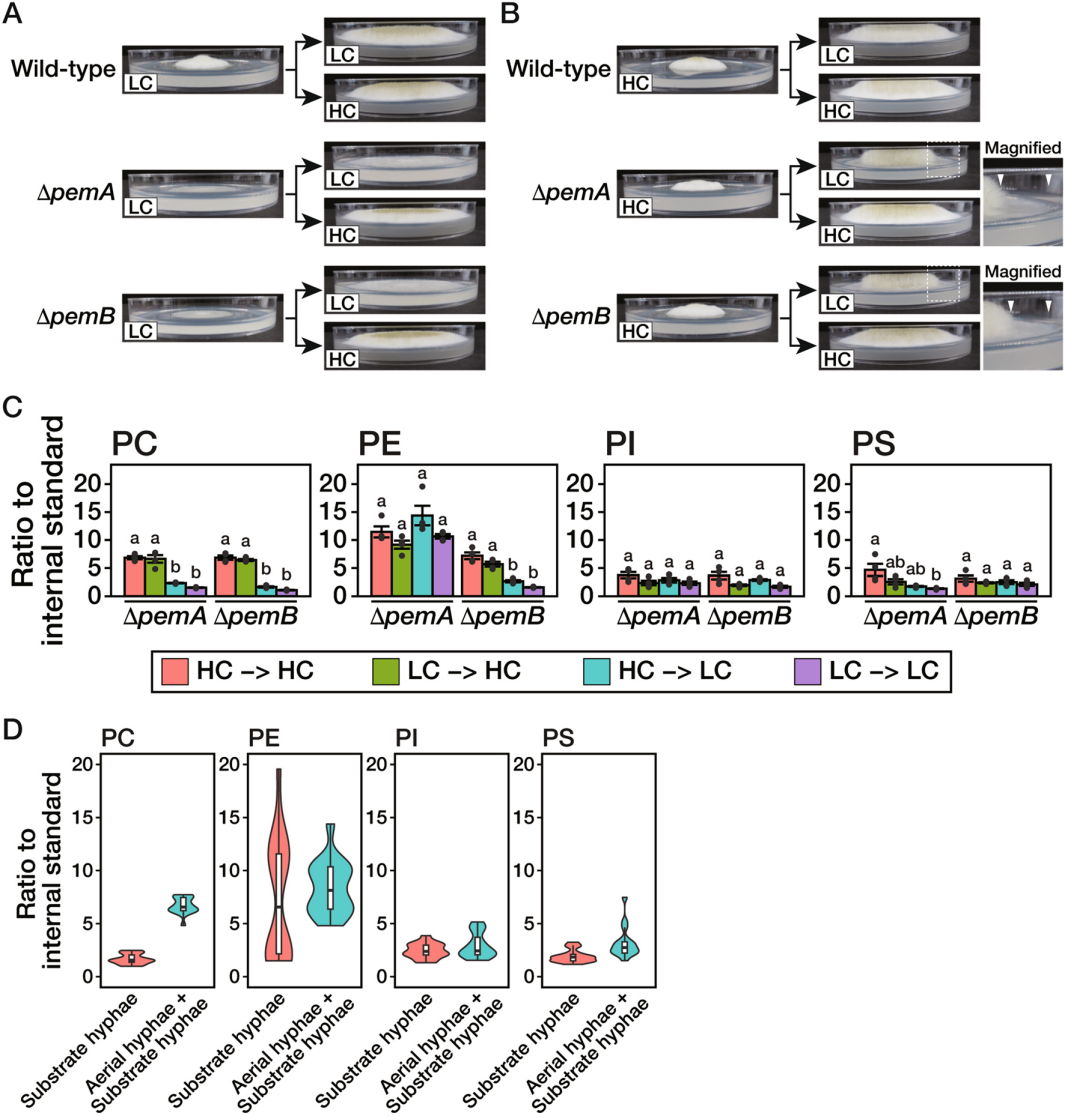
Phospholipid composition in substrate and aerial hyphae. (**A**) The conidia of the wild-type, Δ*pemA*, or Δ*pemB* strain were inoculated on a CD medium containing 1 mM Etn and 5 μM Cho (LC) covered with a cellophane sheet and incubated at 30 °C for 72 h. Then, a cellophane sheet on which mycelia were grown was transferred to the CD medium containing 1 mM Etn and 5 μM Cho (LC) or 1,000 μM Cho (HC) and incubated at 30 °C for 72 h. (**B**) The conidia of the wild-type, Δ*pemA*, or Δ*pemB* strain were inoculated on the “HC” medium covered with a cellophane sheet and incubated at 30 °C for 72 h. Then, a cellophane sheet on which mycelia were grown was transferred to the “LC” or “HC” medium and incubated at 30 °C for 72 h. The dotted rectangles are shown as magnified views. A region of substrate hyphae without aerial hyphae is seen between the *arrowheads*. (**C**) Mycelia of the Δ*pemA* or Δ*pemB* strain were collected from the margin of the colony with a width of 5 mm after the transfer in (**A**) and (**B**). Phospholipids were extracted from these samples and subjected to lipidome analysis. The mean values are depicted as bars (*n* = 4), while dots indicate individual data points. The error bars denote the standard error. Statistically significant differences among phospholipids are indicated by different letters (*P* < 0.01; Tukey–Kramer post-hoc test). (**D**) The lipidomic data in (**C**) were classified into two groups based on those obtained from the mycelia of either substrate hyphae or both substrate and aerial hyphae. Phospholipid level of each group was shown as a box plot and a violin plot.

To ascertain the quantity of PC in the Δ*pemA* and Δ*pemB* strains in the abovementioned experiments, we harvested mycelia cultivated on the CD solid medium containing 5, 20, 100, or 1000 μM Cho covered with a cellophane sheet. We quantified the amounts of PC, PE, PI, and PS (Fig. 3A). We added 1 mM Etn to the medium to supply sufficient PE levels via the Kennedy pathway in this analysis. As Cho concentration was reduced from 1000 to 5 µM, the amounts of PC gradually decreased in the Δ*pemA* and Δ*pemB* strains. The amounts of PE increased significantly as the concentrations of Cho decreased in the Δ*pemA* strain, while those of PE gradually decreased under the same conditions in the wild-type and Δ*pemB* strains. Since PemA, a predicted PE methyltransferase, is present in the Δ*pemB* strain, it is considered that an increase in PE was undetectable due to the conversion of PE to PMME. It is possible that the conversion of PE to PC is suppressed when enough PC is synthesized from exogenous Cho, and that reducing levels of Cho concentration decrease the PE level in the wild-type strain by facilitating the conversion of PE to PC. A correlation analysis was performed to determine how the phospholipid balance was affected by the deletion of *pemA* or *pemB*. The correlation analysis between PC and PE showed a negative correlation for the Δ*pemA* strain and positive correlations for the wild-type and Δ*pemB* strains (Fig. 3B). The decrease in Cho concentration gradually increased the amounts of PI in the Δ*pemA* and Δ*pemB* strains; thus, the PI levels negatively correlated with the PC levels in both strains (Fig. 3B). In addition, the relationships between PI and PE were positive in the Δ*pemA* strain and negative in the Δ*pemB* strain (Fig. 3B). These results suggested that phospholipid balance may be adjusted by the PC, PE, and PI amounts in both strains. On the other hand, the amount of PS was not significantly different in the Δ*pemA* and Δ*pemB* strains (Fig. 3A). Considering that supplementation with 5 μM Cho resulted in drastically low levels of aerial hyphal formation (Fig. 2C–E), our findings suggested that a sufficient amount of PC and/or a low-level amount of PI is required to produce aerial hyphae in *A. oryzae*. As described below in Fig. 4, we proposed that the PC level is a significant factor in inducing the formation of aerial hyphae. In the following experiments, we focused on the characteristics of the Δ*pemA* and Δ*pemB* strains grown on the CD medium with 1000 μM Cho (*High-Cho* medium) and 5 μM Cho (*Low-Cho* medium) as aerial hyphae-formed and -barely formed conditions, respectively.

### Substrate hyphae formed on the *Low-Cho* medium can form aerial hyphae by supplying a sufficient amount of Cho

We investigated whether the substrate hyphae formed under PC-limited conditions still could form aerial hyphae. We incubated the wild-type, Δ*pemA*, or Δ*pemB* strain for three days on a *Low-Cho* medium covered with a cellophane sheet and transferred the sheet to a *Low-Cho* or *High-Cho* medium. Then, it was incubated for additional three days (Fig. 4A). When transferred to the *High-Cho* medium, aerial hyphae were differentiated from a region where substrate hyphae predominantly formed in the Δ*pemA* and Δ*pemB* strains. Furthermore, judging from the coloration of the colonies, these aerial hyphae produced conidia. The formation of aerial hyphae, conidiophores, and conidia was not observed when transferred from a *Low-Cho* medium to the same medium in both strains. In contrast, when the Δ*pemA* or Δ*pemB* strain was transferred from a *High-Cho* medium to a *Low-Cho* medium, aerial hyphae were hardly observed, and only substrate hyphae extended after the transfer (Fig. 4B, between *arrowheads*). Subsequently, we harvested mycelia from the margin of the colony on the plate medium with a width of 5 mm to analyze their phospholipid compositions before and after the transfer. There were differences between the hyphae harvested for lipidomic analyses in Figs. 3 and 4; the entire colony formed after 36 h incubation (Fig. 3), and the region excluding the colony's center formed after 72 h incubation before the medium was changed (Fig. 4). Although slight differences existed, we confirmed that the overall phospholipid compositions were correlated between these conditions (Fig. S7A). In the wild-type strain, slight fluctuations were observed before and after the medium was changed, but only few significant changes were observed (Fig. S7B). In the Δ*pemA* or Δ*pemB* strain, the PC levels significantly increased when transferred to the *High-Cho* medium compared to the *Low-Cho* medium, regardless of the type of medium prior to the transfer (Fig. 4C). These PC levels correlated with the formation of aerial hyphae (Fig. 4A–C).

The lipidomic analysis of the mycelia collected from the entire colonies showed the possibility that PI also correlated with aerial hyphal formation (Figs. 2C and 3A). However, those collected from the margins of the colonies showed no such correlation (Fig. 4C). To clarify the relationship between the aerial hyphal formation and phospholipid levels in hyphae, the data in Fig. 4C were classified into two groups based on those obtained from the mycelia of either only substrate hyphae or both substrate and aerial hyphae regardless of the concentration of Cho in the medium. Analysis of the phospholipid composition exhibited that the distribution of PC level is completely bisected between these two groups (Fig. 4D). In contrast, the distributions of levels of other phospholipids showed overlap between the two groups (Fig. 4D). These findings suggested that PC strongly influences inducing aerial hyphal formation.

### PC synthesized in aerial hyphae is not efficiently utilized near the hyphal tip of substrate hyphae

**Figure 5:**
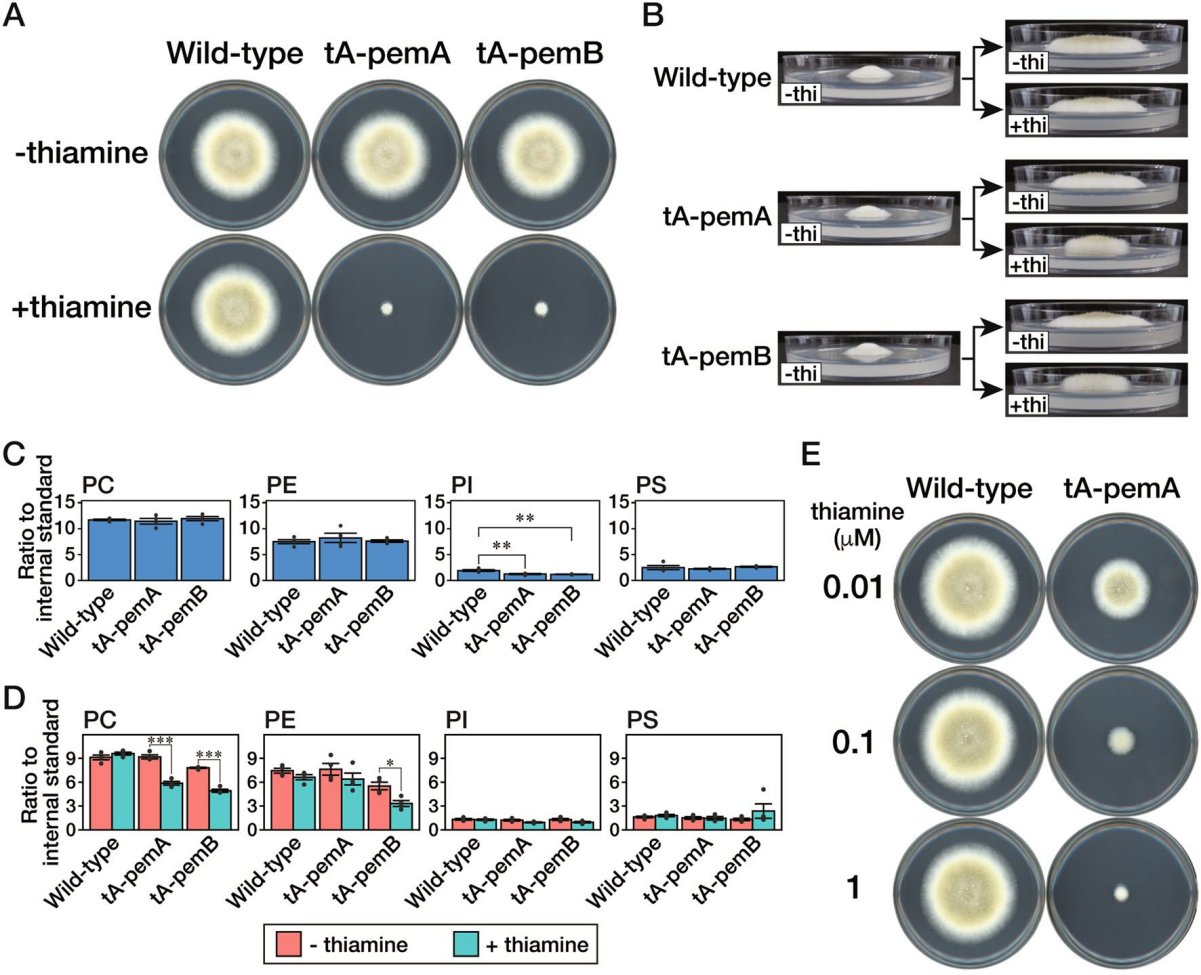
Roles of PC synthesis at the tip of substrate hyphae. (**A**) The conidia of the wild-type, tA-pemA, or tA-pemB strain were inoculated on a CD medium with or without 10 μM thiamine and incubated at 30 °C for 96 h. (**B**) The conidia of the wild-type, tA-pemA, or tA-pemB strain were inoculated on a CD medium covered with a cellophane sheet and incubated at 30 °C for 72 h. Then, a cellophane sheet on which mycelia were grown was transferred to a CD medium with or without 10 μM thiamine (“ + thi”, “-thi”, respectively). (**C**, **D**) Mycelia of the strains were collected from the margin of the colony with a width of 5 mm before the transfer (**C**) or after the transfer (**D**) in (**B**). Phospholipids were extracted from these samples and subjected to lipidome analysis. The mean values are depicted as bars (*n* = 4), while dots indicate individual data points. The error bars denote the standard error. (**C**) Statistically significant differences among phospholipids are indicated by asterisks (**P < 0.01; Tukey–Kramer post-hoc test). (**D**) Statistically significant differences among phospholipids are indicated by asterisks (***P < 0.001, *P < 0.05; Welch’s *t*-test). (**E**) The conidia of the wild-type or tA-pemA strain were inoculated on a CD medium containing 0.01, 0.1, or 1 μM thiamine and incubated at 30 °C for 96 h.

Aerial hyphae were not formed after transferring from a *High-Cho* medium to a *Low-Cho* medium and the amount of PC was low in the substrate hyphae in the *Low-Cho* medium after the transfer, implying that PC in the posterior part of the hyphae was not efficiently transported to the hyphal tips. Therefore, PC must be synthesized near the hyphal tips for their elongation and aerial hyphal formation. In Fig. 4, PC is supplied via the Kennedy pathway in the Δ*pemA* or Δ*pemB* strain since PC synthesis via the *N*-methylation pathway is completely blocked. To investigate the effects of PC synthesis on the *N*-methylation pathway near the hyphal tips, we constructed a strain in which the synthesis of PemA or PemB is regulated via the *thiA* promoter^19^. In this strain, the translation of PemA or PemB is repressed in the presence of thiamine. We named the strain tA-pemA or tA-pemB, respectively. It was demonstrated that these strains showed a similar growth as the wild-type strain when they were grown on the CD medium without thiamine. In contrast, the strains exhibited severe growth retardation in the presence of thiamine (Fig. 5A). When these strains were transferred from a thiamine-free to a thiamine-containing medium, hyphal elongation stopped after the transfer (Fig. 5B), even though PC had been synthesized in a thiamine-free medium before the transfer to the same extent as the wild-type strain (Fig. 5C). However, the aerial hyphae elongated upward (Fig. 5B), possibly due to the de-repression of PemA or PemB synthesis within the upper part of aerial hyphae because of the relatively large distance from a thiamine-containing medium. In fact, in the colony margins containing aerial hyphae after transferred to a thiamine-containing medium, the amount of PC was significantly lower than that in a medium without thiamine (Fig. 5D) but higher than in the area where only substrate hyphae were formed (Figs. 3A and 4C).

The above results suggested that PC is not transported from the aerial hyphae to the hyphal tips of substrate hyphae and that only PC synthesized near the tips of the substrate hyphae could be utilized to elongate hyphal tips. To explore the significance of PC synthesis at the hyphal tips of substrate hyphae, the tA-pemA and tA-pemB strains were grown on the CD medium containing various thiamine concentrations (Figs. 5E and S8). At all concentrations, the phenotype that only substrate hyphae were formed was not observed. Notably, even at very low thiamine concentrations (0.002–0.1 μM), colony area gradually decreased as thiamine concentration increased, while the degree of aerial hyphal formation remained the same. This phenotype was markedly different from that of the Δ*pemA* or Δ*pemB* strain, where the colony area remained unchanged, and only aerial hyphal formation was repressed when the concentration of Cho was decreased. These results suggested that a slight decrease in PemA or PemB reduced the rate of hyphal extension and that PC synthesis at the tips of substrate hyphae is considered an essential factor for tip elongation.

### Substrate hyphae have shorter acyl chains in PC and PE and longer chains in PS

**Figure 6:**
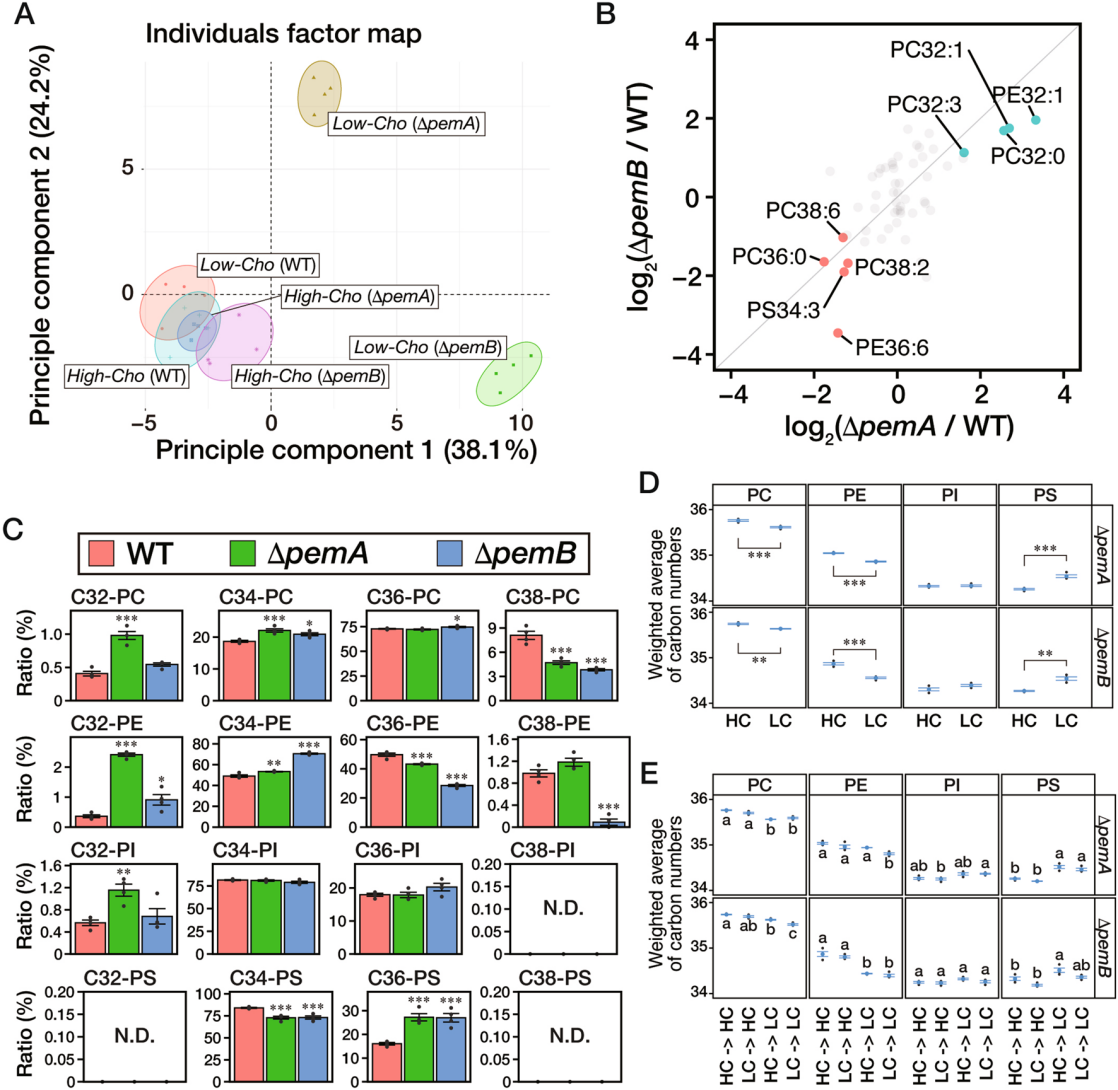
Analysis of phospholipid’s acyl chain composition of substrate and aerial hyphae. (**A**) The data obtained in Fig. 4 were used to calculate the mol% of each acyl chain length. A principal component analysis was performed using the mol% value. (**B**) The ratios of Δ*pemA*/wild-type or Δ*pemB*/wild-type on each acyl-chain moiety in each head group from the data obtained from the mycelia grown on the *Low-Cho* medium were plotted. For phospholipid species that both log_2_(Δ*pemA*/wild-type) and log_2_(Δ*pemB*/wild-type) values are greater than 2 (*blue*) or less than -1 (*red*), the names are given. (**C**) Using the lipidomic data obtained from the mycelia grown on the *Low-Cho* medium, the acyl chain length composition of each phospholipid head group was summed. The mean values are depicted as bars (*n* = 4), while dots indicate individual data points. The error bars denote the standard error. Statistically significant differences compared to the wild-type are indicated by asterisks (***P < 0.001, **P < 0.01, *P < 0.05; Dunnett's test). (**D**, **E**) The data obtained in Fig. 4 were used to calculate the weighted average length of phospholipid acyl chains. *Blue* dots indicate the mean values (*n* = 4), while *gray* dots indicate individual data points. The error bars denote the standard error. HC; *High-Cho* condition, LC; *Low-Cho* condition. (**D**) Asterisks indicate statistically significant differences (***P < 0.001, **P < 0.01; Welch’s two-sample *t*-test). (**E**) Statistically significant differences among phospholipids are indicated by different letters (*P* < 0.01; Tukey–Kramer post-hoc test).

In a previous study using *A. nidulans*, we demonstrated that the PC levels decreased and at the same time, highly unsaturated phospholipids increased in the hyphae at the colony margins compared to those at the relative center of the colony^18^. Since the colony margin is predominantly composed of substrate hyphae, the low levels of PC in this area align with the findings of low levels of PC in substrate hyphae (Fig. 4D). Hence, we addressed whether acyl-chain profiles change between substrate and aerial hyphae. We used lipidomic data derived from mycelia grown on the *Low-Cho* or *High-Cho* medium to calculate the mol% of detailed phospholipid moiety in each head group. We performed a principal component analysis (PCA) using these values (Fig. 6A). The PCA results showed that the Δ*pemA* or Δ*pemB* strains grown on the *Low-Cho* medium were distinct from the other samples, indicating differences in acyl-chain profiles between the substrate and aerial hyphae.

The PCA demonstrated discernible differences in the acyl-chain profiles between the Δ*pemA* and Δ*pemB* strains despite their comparable substrate hyphal formation in the *Low-Cho* medium. We plotted the Δ*pemA*/wild-type or Δ*pemB*/wild-type ratios for each phospholipid moiety under the *Low-Cho* condition to explore the common phospholipid changes between these strains (Fig. 6B). These deletion mutants both exhibited high enrichment of C32:0-PC, C32:1-PC, C32:3-PC, and C32:1-PE; conversely, C36:0-PC, C38:2-PC, C38:6-PC, C36:6-PE, and C34:3-PS were reduced. These findings suggested that substrate hyphae have shorter acyl chains while aerial hyphae have longer ones. Thus, we focused on the phospholipid acyl-chain length by calculating the respective ratios (Fig. 6C). Compared to the wild-type strain, C32-PC, C34-PC, and C32-PE were significantly increased in the Δ*pemA* strain, and C34-PC, C32-PE, and C34-PE were increased in the Δ*pemB* strain. On the other hand, C38-PC and C36-PE exhibited a significant decrease in both strains, and C38-PE drastically decreased in the Δ*pemB* strain. Conversely, PS showed the opposite trend; C34-PS decreased, while C36-PS increased in both strains (Fig. 6C). There was a slight difference in PI, where only C32-PI increased in the Δ*pemA* strain (Fig. 6C). We calculated the weighted average of the lengths in each head group to compare the phospholipid acyl-chain lengths under various conditions. This value decreased in PC and PE but increased in PS under the *Low-Cho* condition, where only substrate hyphae were formed (Fig. 6D). For the results obtained in the experiments where the strains were transferred from a *Low-Cho* to a *High-Cho* medium and vice versa (Fig. 4), we computed the weighted average length of acyl chains on phospholipids extracted from the mycelia. Consequently, we observed that the type of medium after the transfer affected these values, albeit to varying degrees (Fig. 6E). These results suggested that acyl chains in PC and PE were shorter in substrate hyphae than in aerial hyphae, while those in PS were longer. Considering the relatively high concentrations of PC and PE among the main phospholipids in mycelia, *A. oryzae* may shorten the acyl chains, thereby increasing membrane fluidity in substrate hyphae.

## Discussion

Many eukaryotes have PC as a significant component of their membrane lipids, consisting of around 40–60% of total phospholipids^1,20^. In *S. cerevisiae*, the mechanisms and regulations involved in phospholipid synthesis have been studied extensively^20,21^. Although some progress has been made in understanding the PC synthesis pathways and the phenotypic changes caused by perturbations in filamentous fungi, the physiological role of PC is yet to be elucidated. In this study, the amount of PC was artificially altered in the filamentous fungus *A. oryzae*. The study revealed that the intracellular content of PC is crucial for the differentiation process from substrate to aerial hyphae. Although several studies measured PC concentrations in *A. nidulans* and *F. graminearum* when different concentrations of Cho were added to the media^22,23^, this is the first report to demonstrate that aerial hyphae differentiation is regulated by the amount of PC in filamentous fungi.

In *A. oryzae*, Δ*pemA* and Δ*pemB* strains showed severe growth retardation in a minimal medium (Fig. 2A). In *A. niger*, the *PEM1* ortholog, *cho2,* deletion strain did not grow on a minimal medium, while the *PEM2* ortholog, *opi3,* deletion strain grew poorly on the same medium^14^. These results suggested that Cho2 of *A. niger* could perform the Opi3 function, and that there are some functional differences between Cho2 of *A. niger* and PemA of *A. oryzae*. Cho2 of *A. niger* is a 1077-amino acid protein, while PemA of *A. oryzae* is a 971-amino acid protein. Comparing these two proteins, PemA has a large deletion in its C-terminal region (Fig. S9A, *Region 3*). Additionally, two regions of *A. oryzae* PemA were deleted in *A. niger* Cho2 (Fig. S9A, *Regions 1* and *2*). These regions are thought to be located at the surfaces of the enzymes, according to the predicted structures by AlphaFold2^24^ (Fig. S9B), and their deduced domain organizations are quite similar (Figs. S2B and S9C). Since the catalytic mechanism of Cho2 is still unknown even in *S. cerevisiae*, it is currently unclear whether these differences in amino acid sequences affect their catalytic activities.

Balloon-like structures were formed from the hyphal tips to the subapical regions when culturing the Δ*pemA* or Δ*pemB* strain on a medium without Cho (Fig. 2B). Previous studies in *A. nidulans* showed a correlation between PC levels and a chitin synthase activity was observed^23^. A deficiency in PC synthesis led to a shift from polarized to depolarized growth in *A. nidulans*^25^. The balloon formation phenotypes were also observed in the Δ*choC* strains of *A. nidulans* and *P. microspore*^10,13^. These results strongly supported that a higher PC level is required for the chitin synthesis and the polarity establishment in filamentous fungi. Furthermore, since some deletion mutants of chitin synthase-encoding genes in *A. nidulans* and *A. oryzae* formed balloon-like structures^26,27^, it is possible that their formation in the Δ*pemA* and Δ*pemB* strains is caused by malfunctions of these chitin synthases.

Previous studies have shown that the deletion of specific genes involved in autophagy leads to the failure to form aerial hyphae in *A. oryzae*^28,29^. The defects in aerial hyphal formations with the deletion of autophagy-associated genes were also observed in other filamentous ascomycete fungi, such as *Arthrobotrys oligospora*^30^, *Bipolaris maydis*^31^, *Botrytis cinerea*^32–34^, *Cochliobolus heterostrophus*^35^, *F. graminearum*^36–38^, *Fusarium verticillioides*^39^, *Magnaporthe oryzae*^40^, and *Podospora anserina*^41^. It is currently unknown how these genes contribute to aerial hyphal formation, and the precise mechanisms that induce these phenotypic changes have yet to be determined. Recently, studies in *S. cerevisiae* suggested that de novo-synthesized PC is incorporated into the autophagosomes^42^, and that de novo PC is required for autophagosome formation^43^. Hence, when the Δ*pemA* and Δ*pemB* strains are cultured in a *Low-Cho* medium, it is likely that their autophagy does not function properly due to the lack of PC. However, it is possible that autophagy supplies the substrates of the enzymes involved in the Kennedy pathway during the formation of aerial hyphae in the wild-type strain (Fig. 1), and that the autophagy deficiency may cause depletion of PC, resulting in the absence of aerial hyphal formation in the autophagy-deficient strains. The fact that most of genes involved in the Kennedy pathway were upregulated during aerial hyphae and conidiophore formation in the wild-type strain supports this idea (Fig. S4). A closer examination of the relationships between PC and autophagy in filamentous fungi could unravel the intricate mechanism behind aerial hyphal formation.

The synthesis of PC is achieved via the Kennedy or CDP-DAG pathway, as shown in Fig. S1. In *A. oryzae*, the phenotype remained unchanged even if PC was obtained via either pathway as long as enough PC was supplied (Fig. 2A, Wild-type (−) vs. Δ*pemA* (+ Cho) or Δ*pemB* (+ Cho)). On the other hand, when the PC levels in hyphae were insufficient, the phenotypes varied based on the PC synthesis pathway. When limited amounts of PC were/was synthesized via the Kennedy pathway, only substrate hyphae were formed and elongated (Fig. 2A). In contrast, similar phenotypes were not observed when PC was supplied via the *N*-methylation pathway (Fig. 5E). This phenotypic difference could be caused by the difference of the pathways that synthesize PC. However, comparing the intracellular PC levels between both conditions (Fig. 3A and 4C vs. Fig. 5D), PC level of the Δ*pemA* or Δ*pemB* strain grown on the medium containing 5 μM Cho was lower than that of tA-pemA or tA-pemB strain grown on the medium containing thiamine. Thus, it is possible that aerial hyphal formation is dependent on the intracellular concentration of PC and not dependent on the PC synthesis pathway. In the tA-pemA or tA-pemB strain, where the translational repression of *thiA* is leaky even on the medium containing sufficient amount of thiamine^44^, the aerial hyphae could extend slightly due to the leaky production of PemA or PemB. The development of alternative approaches that do not use the *thiA* promoter is required to clarify the cause of these phenotypic differences between these deletion mutants and conditional mutants using *thiA* promoter. In *S. cerevisiae*, Ept1 and Cpt1 localize to the ER (endoplasmic reticulum) and/or Golgi^2,21^, while Pem1 and Pem2 predominantly localize to the ER^21^. Recent researches suggested that active vesicle transport is essential for rapidly extending hyphal tips^45,46^, with the Golgi playing a crucial role in the process. This raises the possibility that PC synthesis via the Kennedy pathway may be crucial for the rapid extension of hyphae.

This study demonstrated that mycelia grown on either the *Low-Cho* or *High-Cho* medium had different trends in their phospholipid acyl chain lengths (Fig. 6B–D). There could be a requirement for longer acyl chains to facilitate aerial hyphal formation. However, the possibility that the substrate hyphae grown on the *High-Cho* medium would have the different acyl chain composition from those grown on the *Low-Cho* medium could not be excluded. To determine whether phospholipids with longer acyl chains are found only in aerial hyphae or in both aerial and substrate hyphae grown on the *High-Cho* medium, it is necessary to establish a method for measuring the acyl chain length in each of the two types of hyphae. The Δ*pemA* and Δ*pemB* strains exhibited similar phenotypes when cultured in a *Low-Cho* medium. However, significant differences were observed in their phospholipid composition (Fig. 6A and C). Since the transcriptome profiles are dramatically different between the Δ*cho2* and Δ*opi3* strains of *A. niger*^14^, it is possible that phospholipid compositions could become diverse due to the global changes in the transcriptome profiles in *A. oryzae*. A more comprehensive lipidome and transcriptomic analysis could help understand the regulation of phospholipid balance in filamentous fungi.

### Experimental procedures

#### Strains, growth media, and transformations

Strains used in this study are listed in Table S2. The R40Δku5-2^47^ was used as a wild-type strain, and the R40Δku5-2ΔpyrG (Katayama unpublished) was constructed by deleting the *pyrG* gene of R40Δku5-2 and was used as a parental strain for transformation. The *A. oryzae* strains were incubated at 30 °C in a modified Czapek-Dox (CD) medium (30.0 g/L glucose, 3.0 g/L sodium nitrate, 1.0 g/L dipotassium phosphate, 0.5 g/L magnesium sulfate heptahydrate, 0.5 g/L potassium chloride, 1.0 mg/L iron(II) sulfate heptahydrate, 8.8 mg/L zinc sulfate heptahydrate, 0.4 mg/L copper(II) sulfate pentahydrate, 0.15 mg/L manganese(II) sulfate tetrahydrate, 0.1 mg/L sodium tetraborate decahydrate, 0.05 mg/L ammonium molybdate tetrahydrate, and 1 μL/L sulfuric acid). 1.5%(w/v) agar was added to prepare a solid medium. Ten μM thiamine, 10 mM uridine, 10 mM uracil, 1 mM Etn, and 1 mM Cho were added to the CD medium when necessary.

To prepare a stock conidia solution, the *A. oryzae* strain was incubated on the polypepton dextrin (PD) solid medium^48^ at 30 °C for seven days, after which 8 mL of 0.01% (v/v) Tween 80 was poured into the plate and conidia were scraped off with a cell spreader. The obtained solution was filtered with Miracloth (475855; Merck Millipore, Burlington, MA).

In solid media covered with or without a cellophane sheet, 1.0 × 10^3^ conidia were spotted and incubated at 30 °C. For solid medium transfer, a cellophane sheet on which mycelia were grown was transferred to a new medium. In liquid media, 2.0 × 10^7^ conidia were inoculated and incubated at 30 °C and 120 rpm. To extract total RNAs for 5′-RACE, 1.0 × 10^7^ conidia of the NSPlD1 strain^49^ were incubated at 30 °C overnight in 50 mL of malt medium (30 g/L malt extract, 3 g/L yeast extract, 5 g/L glucose, 2.44 g/L uridine, pH 6.0). The transformations of *A. oryzae* and *Escherichia coli* were performed as described previously^50–52^.

#### Construction of plasmids and A. oryzae strains

Plasmids and primers used in this study are listed in Tables S3 and S4, respectively.

We constructed the plasmid p18-ΔpemA-pG as follows: a fragment containing an upstream (−804 to −1 bp) or a downstream (+ 3170 to +3972 bp) region of *pemA* was amplified from the RIB40 genomic DNA by polymerase chain reaction (PCR) using primers TSAP184 and TSAP185 or TSAP187 and TSAP188, respectively. The amplified fragments were cloned into pUC18 by SLiCE^53–55^, yielding p18-ΔpemA. The fragment containing *pyrG* (− 1004 to + 1394 bp) was amplified from the RIB40 genomic DNA by PCR using primers TSAP1 and TSAP2, and the amplified fragment was cloned into pUC18, yielding p18-pG. A fragment containing an upstream and a downstream region of *pemA* was amplified from the p18-ΔpemA by PCR using primers TSAP237 and TSAP238, and a *pyrG* fragment was amplified from the p18-pG using primers TSAP132 and TSAP15. These fragments were ligated by SLiCE, yielding the plasmid p18-ΔpemA-pG.

We constructed the plasmid p18-ΔpemB-pG as follows: a fragment containing an upstream (− 804 to − 1 bp) or a downstream (+ 985 to + 1784 bp) region of *pemB* was amplified from the RIB40 genomic DNA by PCR using primers TSAP189 and TSAP190 or TSAP192 and TSAP193, respectively. The amplified fragments were cloned into pUC18 by SLiCE, yielding p18-ΔpemB. A fragment containing an upstream and a downstream region of *pemB* was amplified from the p18-ΔpemB by PCR using primers TSAP239 and TSAP240, and a *pyrG* fragment was amplified from the p18-pG using primers TSAP132 and TSAP15. These fragments were ligated by SLiCE, yielding the plasmid p18-ΔpemB-pG.

To construct a strain in which the expression of *pemA* or *pemB* is regulated by *thiA* promoter (*thiA*p), a fragment containing the promoter region of *thiA* (*thiA*p: − 1288 to − 1 bp) was amplified from the RIB40 genomic DNA by PCR using primers TSAP25 and TSAP34. A *pyrG* fragment was amplified from p18-pG using primers TSAP132 and TSAP15. The *thiA*p and *pyrG* fragments were cloned into pUC18 by SLiCE, yielding p18-pG-tA. The fragments containing an upstream region of *pemA* (− 1093 to − 1 bp), a part of *pemA* coding region (+ 1 to + 1012 bp), an upstream region of *pemB* (− 1071 to − 1 bp), or the *pemB* coding region and a downstream region (+ 1 to + 1049 bp) were amplified from the RIB40 genomic DNA by PCR using primers TSAP103 and TSAP104, TSAP106 and TSAP126, TSAP108 and TSAP109, or TSAP111 and TSAP112, respectively. The fragments of the upstream and the Open Reading Frame (ORF)-encoding regions of *pemA* or *pemB* and a *pyrG*-*thiA*p fragment, amplified from p18-pG-tA using primers TSAP1 and TSAP124, were cloned into pBlueScript II SK ( +) by SLiCE, yielding the plasmid pBS-pG-tA-pemA or pBS-pG-tA-pemB, respectively.

The DNA fragments were amplified by PCR from p18-ΔpemA-pG or p18-ΔpemB-pG using primers CGUP1_UF1 and CGUP2_UR1, or from pBS-pG-tA-pemA or pBS-pG-tA-pemB using primers CGUP3_UF2 and CGUP4_UR2. Each fragment was transformed into the R40Δ ku5-2ΔpyrG strain. Several transformants were selected and confirmed by genomic PCR. The strains were designated as Δ*pemA*, Δ*pemB*, tA-pemA, or tA-pemB, respectively.

#### 5*′*-Rapid amplification of cDNA ends (RACE)

The harvested mycelia were frozen in liquid nitrogen and ground using a mortar and pestle. The broken mycelia and 0.5 mm zirconia balls were added to a microtube. The mycelia were completely broken using a Multi-Beads Shocker (Yasui Kikai, Osaka, Japan) at 2700 rpm, 60 s, and repeated three times. According to the manufacturer’s instructions, the total RNA was extracted from the broken mycelia using ISOGEN II (317-07363; NIPPON GENE, Tokyo, Japan). 5'-Rapid amplification of cDNA ends (5′-RACE) was performed using the extracted RNAs and the SMARTer RACE 5′/3′ Kit (Z4858N, TaKaRa Bio, Shiga, Japan), according to the manufacturer’s instructions, with primers pemA_GSP_R and pemA_NGSP_R for 5′-RACE of *pemA*, pemB_GSP_R and pemB_NGSP_R for 5′-RACE of *pemB* (Table S4).

#### Microscopic observations

The hyphae were observed using a BX52 microscope (Evident, Tokyo, Japan) equipped with a CCD camera DP80 (Evident). The images captured by DP80 were acquired and processed using the cellSens software (Evident). Substrate hyphae growing on and in the solid medium were observed using a video microscope JVA-0756 (Wraymer, Osaka, Japan), equipped with a CMOS camera WRAYCAM-NOA2000 (Wraymer). The images captured by WRAYCAM-NOA2000 were acquired and processed using the Spectman software (Wraymer).

#### Lipid extraction and the targeted lipidomic analysis

The mycelia formed on a solid medium covered with a cellophane sheet were collected using a spatula. The harvested mycelia were lyophilized using FLEXI DRY (Kinetics, Stone Ridge, NY), and their dry cell weight was measured. This was followed by lipid extraction and the targeted lipidomic analysis of extracted lipids, as described previously^18^. Briefly, the lyophilized mycelia were broken using a Micro Smash MS-100R (Tomy Seiko, Tokyo, Japan), and lipids were extracted from the broken mycelia using the BUME method^56^. As internal standards, 1 nmol of 15:0–18:1-d7-PC, 1 nmol of 15:0–18:1-d7-PE, 1 nmol of 15:0–18:1-d7-PS, and 1 nmol of 15:0–18:1-d7-PI (Avanti Polar Lipids, Alabaster, AL) were added during the extraction. The extracted lipids were dissolved in equal volumes of methanol and acetonitrile and analyzed according to Nakao et al.^57^. The individual lipid contents were calculated by relating the peak area of the analyte to the peak area of the corresponding internal standard and these values were referred to as the "ratio to internal standard". To clarify sample-to-sample variation in each head group, Z scores were calculated within the experimental group using the sum of the "ratio to internal standard" in each head group.

#### Quantitative reverse transcription PCR (RT-qPCR)

Harvested mycelia were frozen in liquid nitrogen in a microtube and disrupted using a Freeze Crusher μT-48 (Taitec) with a 48pcs-Holder for μT-48 (TH-0248 T, Taitec) at 1000 rpm, 15 s 3 times. The metal crusher was removed, and total RNAs were extracted using a Maxwell RSC simplyRNA Tissue Kit (AS1340; Promega K.K., Tokyo, Japan). Reverse transcription of RNA was performed using a ReverTra Ace qPCR RT Master Mix with gDNA Remover (FSQ-301; Toyobo, Osaka, Japan). Quantitative PCR was performed using the 7300 Real-Time PCR System (Applied Biosystems, Waltham, MA) and THUNDERBIRD Next SYBR™ qPCR Mix (QPX-201; Toyobo, Osaka, Japan). The ΔΔCt method was utilized to compare the expression of each gene, with primers TSAP230 and TSAP231 for *pemA*, TSAP212 and TSAP213 for *pemB*, TSAP708 and TSAP709 for *ekiA*, TSAP208 and TSAP209 for *ectA*, TSAP712 and TSAP713 for *eptA*, TSAP705 and TSAP706 for *ckiA*, TSAP204 and TSAP205 for *cctA*, and TSAP702 and TSAP703 for *cptA*, respectively (Table S4). A gene encoding histone H2B was utilized as a house-keeping gene with primers QP037 and QP038.

#### Data analysis, statistical analysis, and visualizations of numerical data

All analyses were performed using R Statistical Software (v4.2.3; R Core Team, 2023) and RStudio (v2023.03.0 + 386; Posit team, 2023), with R packages: *tidyverse* (v1.3.2; Wickham H, 2019)^58^, *multcomp* (v1.4.23; Hothorn T et al., 2008), and *readxl* (v1.4.0; Wickham H, Bryan J, 2022). The visualization of numerical data was performed using R packages; *ggplot2* (v3.4.1; Wickham H, 2016), *factoextra* (v1.0.7; Kassambara A, Mundt F, 2020), *GGally* (v2.1.2; Schloerke B et al., 2021), *ggrepel* (v0.9.3; Slowikowski K, 2023), and *lemon* (v0.4.6; Edwards S, 2022).

### Supplementary Information

Supplementary Information.

## Data Availability

All data generated or analyzed during this study are included in this published article (and a Supplementary Information file).
