# Supplementary material for: Phosphatidylcholine levels regulate hyphal elongation and differentiation in the filamentous fungus Aspergillus oryzae
Source: Sci Rep. 2024 May 22;14:11729. doi: 10.1038/s41598-024-62580-4 (PMC11111764; doi:10.1038/s41598-024-62580-4)
Supplement: Supplementary file 1 — Supplementary Information. [file 41598_2024_62580_MOESM1_ESM.pdf]

Title

Phosphatidylcholine levels regulate hyphal elongation and differentiation in the filamentous fungus *Aspergillus oryzae*

Running Title

PC regulates hyphal differentiation in *Aspergillus oryzae*

Authors

Tetsuki Suzawa, Ryo Iwama, Ryouichi Fukuda, Hiroyuki Horiuchi

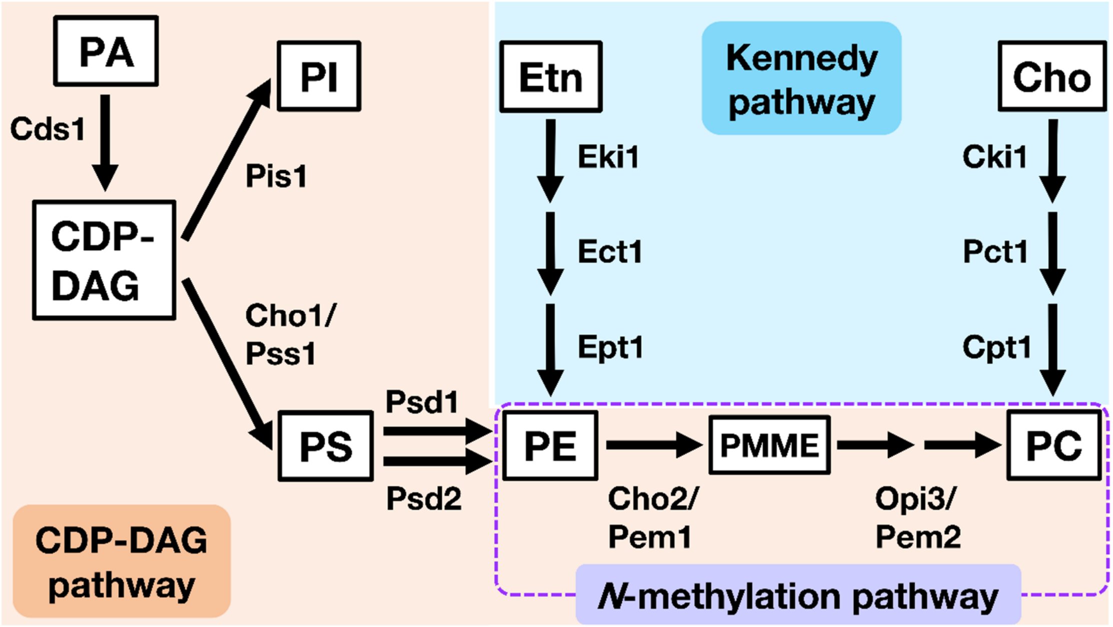

Fig. S1. Phospholipid synthesis pathway in *S. cerevisiae*.

See text for detail.

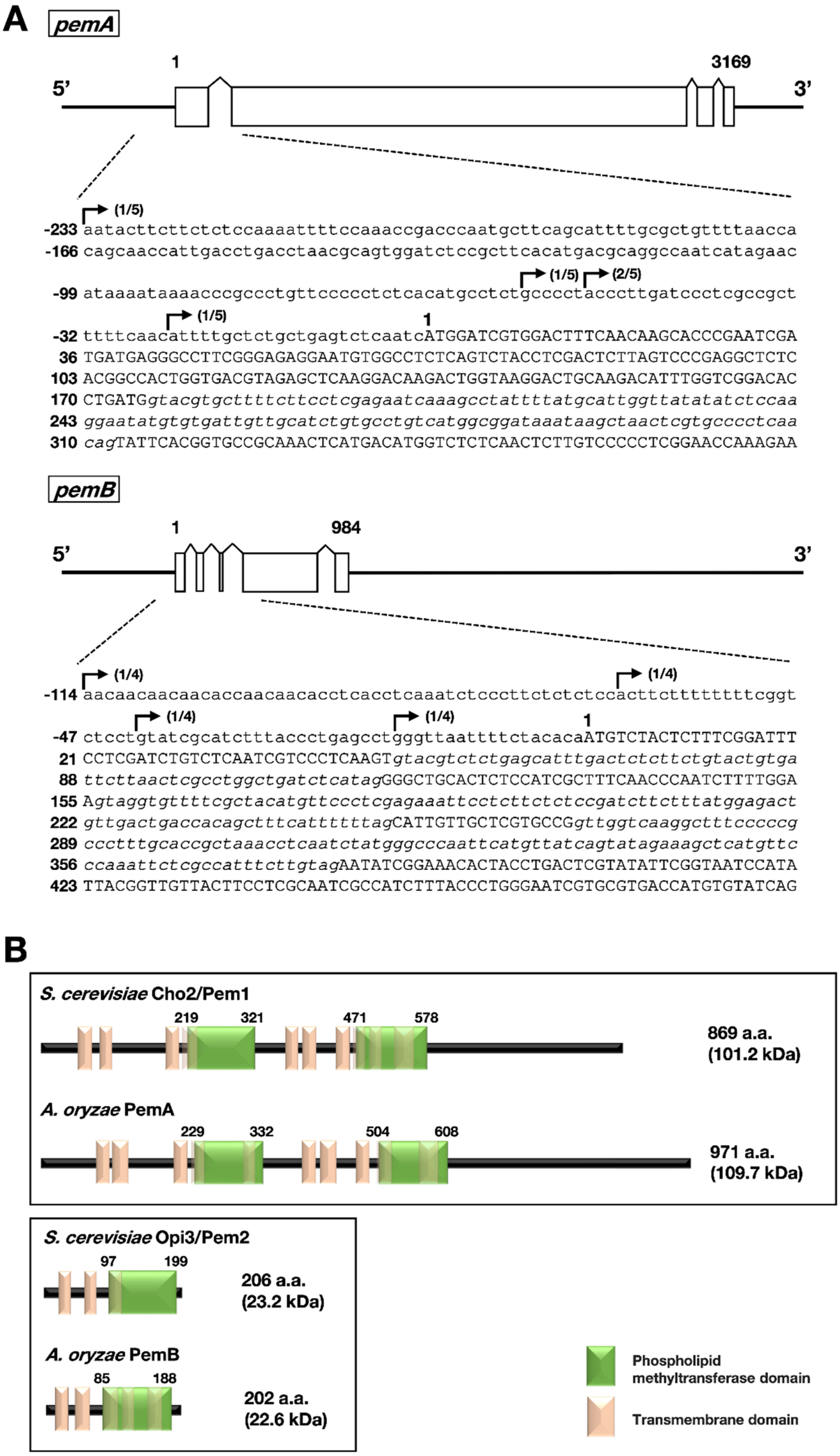
A
pemA
1
3169
5'
3'
(1/5)
-233 aatacttcttctctccaaaattttccaaaccgacccaatgcttcagcattttgcgctgttttaacca
-166 cagcaaccattgacctgacctaacgcagtggatctccgcttcacatgacgcaggccaatcatagaac
(1/5) (2/5)
-99 ataaaataaaacccgccctgttccccctctcacatgcctctgcccctacccttgatccctcgccgct
(1/5) 1
-32 ttttcaacatttttgctctgctgagtctcaatcATGGATCGTGGACTTTCAACAAGCACCCGAATCGA
36 TGATGAGGGCCTTCGGGAGAGGAATGTGGCCTCTCAGTCTACCTCGACTCTTAGTCCCGAGGCTCTC
103 ACGGCCACTGGTGACGTAGAGCTCAAGGACAAGACTGGTAAGGACTGCAAGACATTTGGTCGGACAC
170 CTGATGgtacgtgcttttcttcctcgagaatcaaagcctattttatgcattggttatatatctccaa
243 ggaatatgtgtgattgttgcatctgtgcctgtcatggcggataaataagctaactcgtgcccctcaa
310 cagTATTCACGGTGCCGCAAACTCATGACATGGTCTCTCAACTCTTGTCCCCCTCGGAACCAAAGAA
pemB
1
984
5'
3'
(1/4) (1/4)
-114 aacaacaacaacaccaacaacacctcacctcaaatctcccttctctctccacttcttttttttcggt
(1/4) (1/4) 1
-47 ctcctgtatcgcatctttaccctgagcctgggttaattttctacacaATGTCTACTCTTTCGGATTT
21 CCTCGATCTGTCTCAATCGTCCCTCAAGTgtacgtctctgagcatttgactctcttctgtactgtga
88 ttcttaactcgcctggctgatctcatagGGGCTGCACTCTCCATCGCTTTCAACCCAATCTTTTGGA
155 Agtaggtgttttcgctacatgttccctcgagaaattcctcttctctccgatcttctttatggagact
222 gttgactgaccacagctttcatttttttagCATTGTTGCTCGTGCCGgttggtcaaggctttcccccg
289 ccctttgcaccgctaaacctcaatctatgggcccaattcatgttatcagtatagaaagctcatgttc
356 ccaaattctcgccatttcttgtagAATATCGGAAACACTACCTGACTCGTATATTCGGTAATCCATA
423 TTACGGTTGTTACTTCCTCGCAATCGCCATCTTTACCCTGGGAATCGTGCGTGACCATGTGTATCAG
B
S. cerevisiae Cho2/Pem1
219 321 471 578
869 a.a.
(101.2 kDa)
A. oryzae PemA
229 332 504 608
971 a.a.
(109.7 kDa)
S. cerevisiae Opi3/Pem2
97 199
206 a.a.
(23.2 kDa)
A. oryzae PemB
85 188
202 a.a.
(22.6 kDa)
Phospholipid methyltransferase domain
Transmembrane domain

Fig. S2. 5'-RACE analyses of *pemA* and *pemB*, and domain organizations of PemA and PemB.

(A) Transcription start sites are indicated by right-pointing arrows. The number at the right of the arrow indicates the number of reads to the total number of reads. The "1" indicates the first position of the estimated start codon. Introns are shown in italic lowercase letters. (B) The deduced domain organizations of *S. cerevisiae* Pem1, *A. oryzae* PemA, *S. cerevisiae* Pem2, and *A. oryzae* PemB by InterPro (https://www.ebi.ac.uk/interpro/).

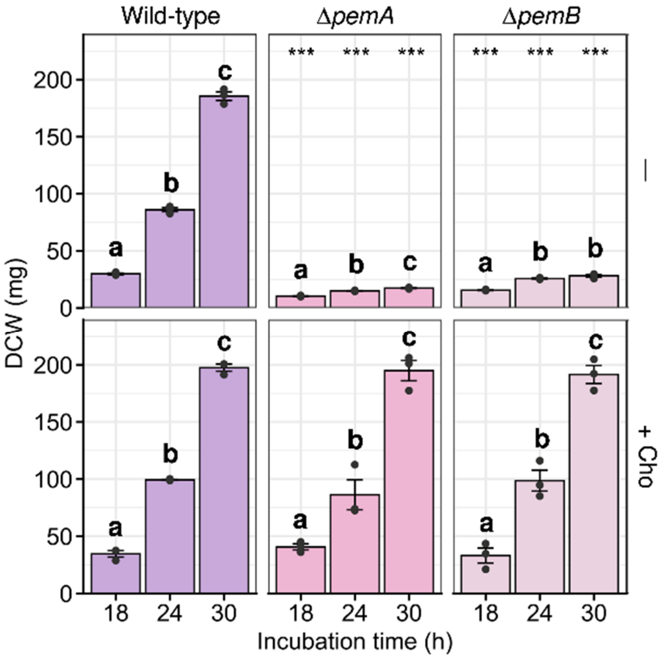

Fig. S3. Changes in DCW of each strain cultured in the CD liquid medium.

The conidia of the wild-type, Δ*pemA,* or Δ*pemB* strain were inoculated into the CD liquid medium containing 1 mM Etn (–) or that containing 1 mM Etn and 1 mM Cho (+ Cho) and incubated at 30°C. The bars, the dots, and the error bars indicate the mean (n = 3), individual data, and the standard error, respectively. Statistically significant differences among DCWs under the same culture conditions and the same strains are indicated by different letters ($P < 0.05$; Tukey's HSD test). Statistically significant differences among DCWs under the same culture conditions and at the same incubation time are indicated by asterisks (***$P < 0.001$; Dunnett's test).

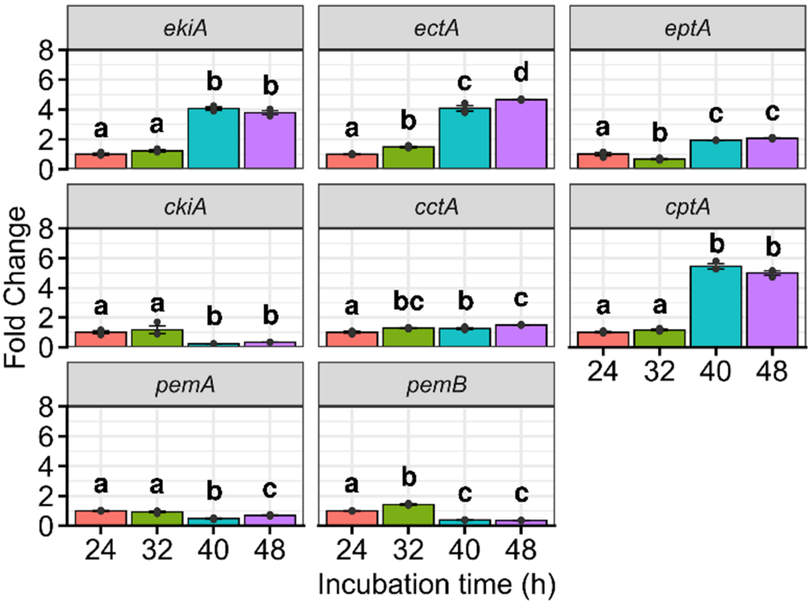

Fig. S4. Changes in the transcript levels during the formation of aerial hyphae and conidiophores. Total RNA was extracted from the colonies, and the mRNA levels of genes related to PC synthesis were quantified. The fold changes were calculated compared to mycelia incubated for 24 h. The bars, the dots, and the error bars indicate the mean ($n = 3$), individual data, and the standard error, respectively. Statistically significant differences among fold changes of the same genes are indicated by different letters ($P < 0.05$; Tukey's HSD test).

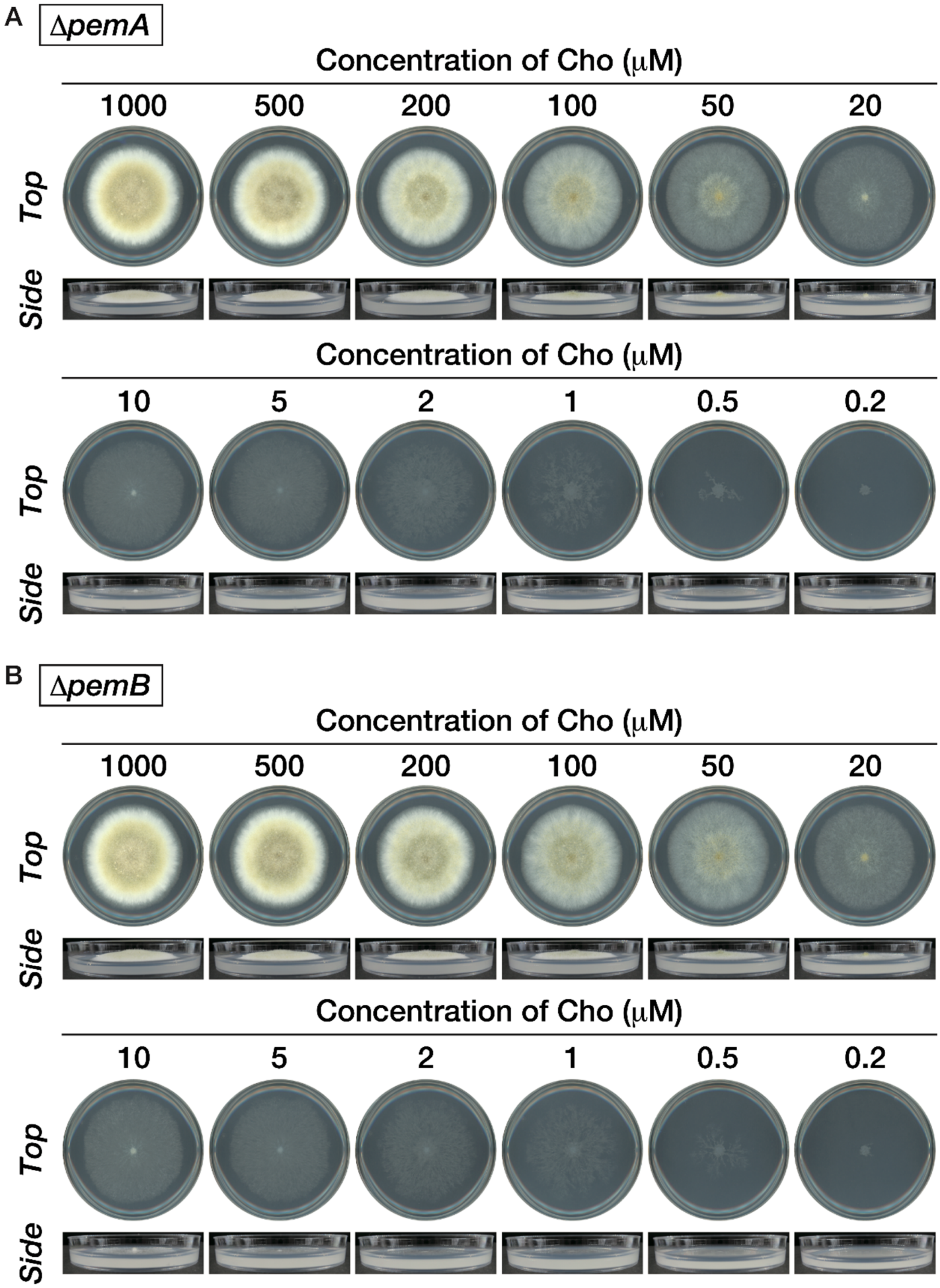

Fig. S5. Growth of the Δ*pemA* or Δ*pemB* strain at different concentrations of Cho.

(A, B) The conidia of the Δ*pemA* (A) and Δ*pemB* (B) were inoculated on a CD medium containing 1 mM Etn and Cho at different concentrations. Then, they were incubated at 30°C for 96 h. Top

and side views of the colonies were shown on the plates containing each concentration of Cho. The Δ*pemA* colonies at 5, 20, 100, and 1,000 μM Cho were also shown in Fig. 2C.

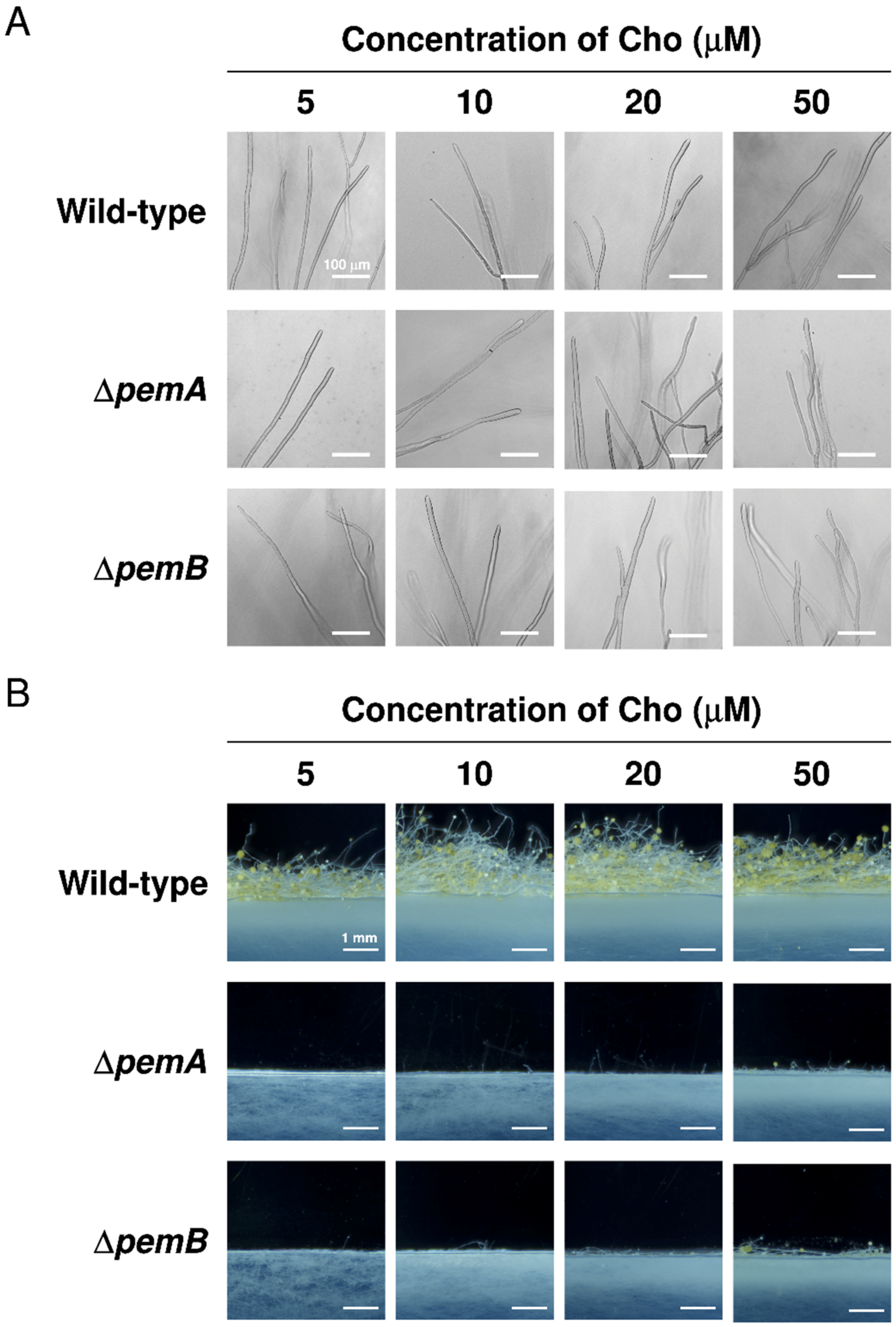

Fig. S6. Hyphal morphology of the deletion mutant of *pemA* or *pemB* on the media containing different concentrations of Cho.

(A) The conidia of the wild-type, Δ*pemA,* or Δ*pemB* strain were inoculated on the CD medium containing 1 mM Etn and 5, 10, 20, or 50 μM Cho and incubated at 30°C for 96 h. The hyphae at the edge of the colonies were observed. (B) The aerial hyphae and substrate hyphae were observed.

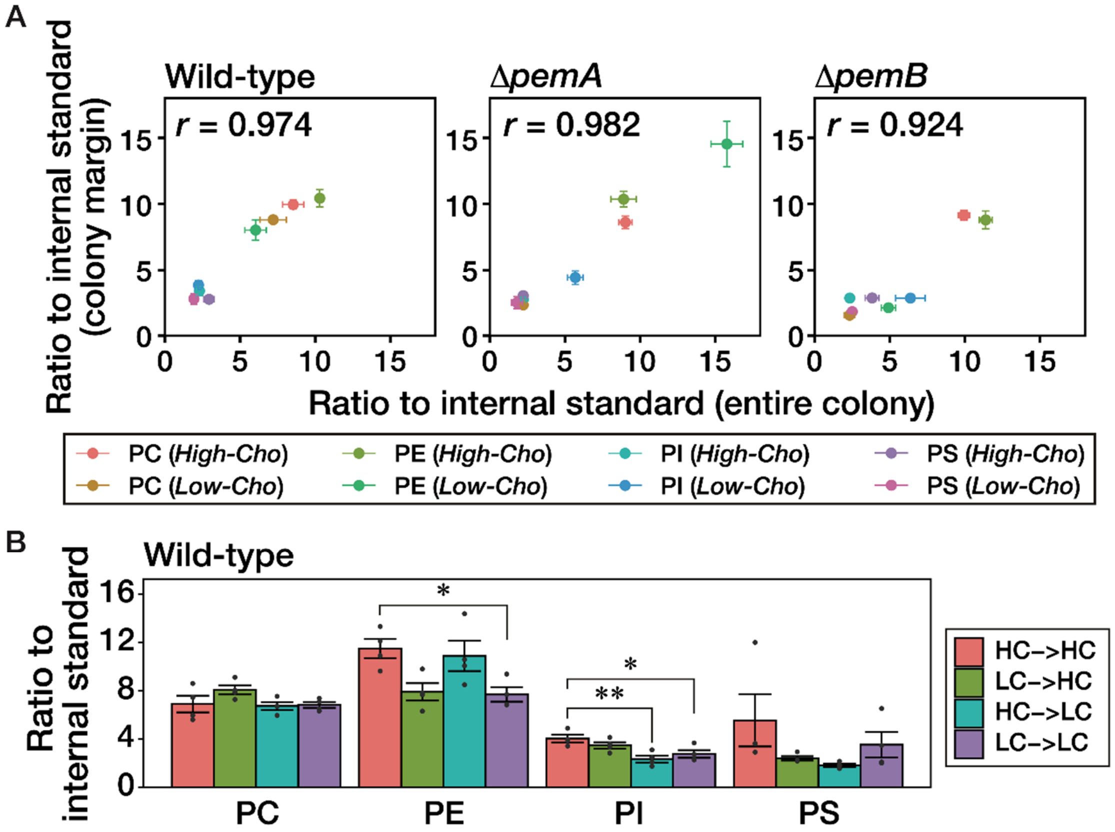

Fig. S7. Validity of phospholipid analysis in medium-transferring experiments (related to Fig. 4C).

(A) The conidia of the wild-type, Δ*pemA*, or Δ*pemB* were inoculated on a CD medium containing 1 mM Etn and 5 μM Cho (LC) or 1,000 μM Cho (HC) covered with a cellophane sheet and incubated at 30°C for 72 h. Phospholipids were extracted from mycelia harvested from the margin of the colony with a width of 5 mm and subjected to lipidome analysis. The obtained data (colony margin) and the data of 5 μM Cho and 1,000 μM Cho derived from Fig. 3A (entire colony) were plotted; dots indicate the mean and error bars represent S.E. The values shown indicate the correlation coefficient in each strain. (B) Mycelia of the wild-type strain were harvested as described in (A) after the transfer in Fig. 4A and B. Phospholipids were extracted from these samples and subjected to lipidome analysis. The mean values are depicted as bars ($n = 4$), while dots indicate individual data points. The error bars denote the standard error. Statistically significant differences are indicated by asterisks (**$P < 0.01$, *$P < 0.05$; Tukey-Kramer post-hoc test).

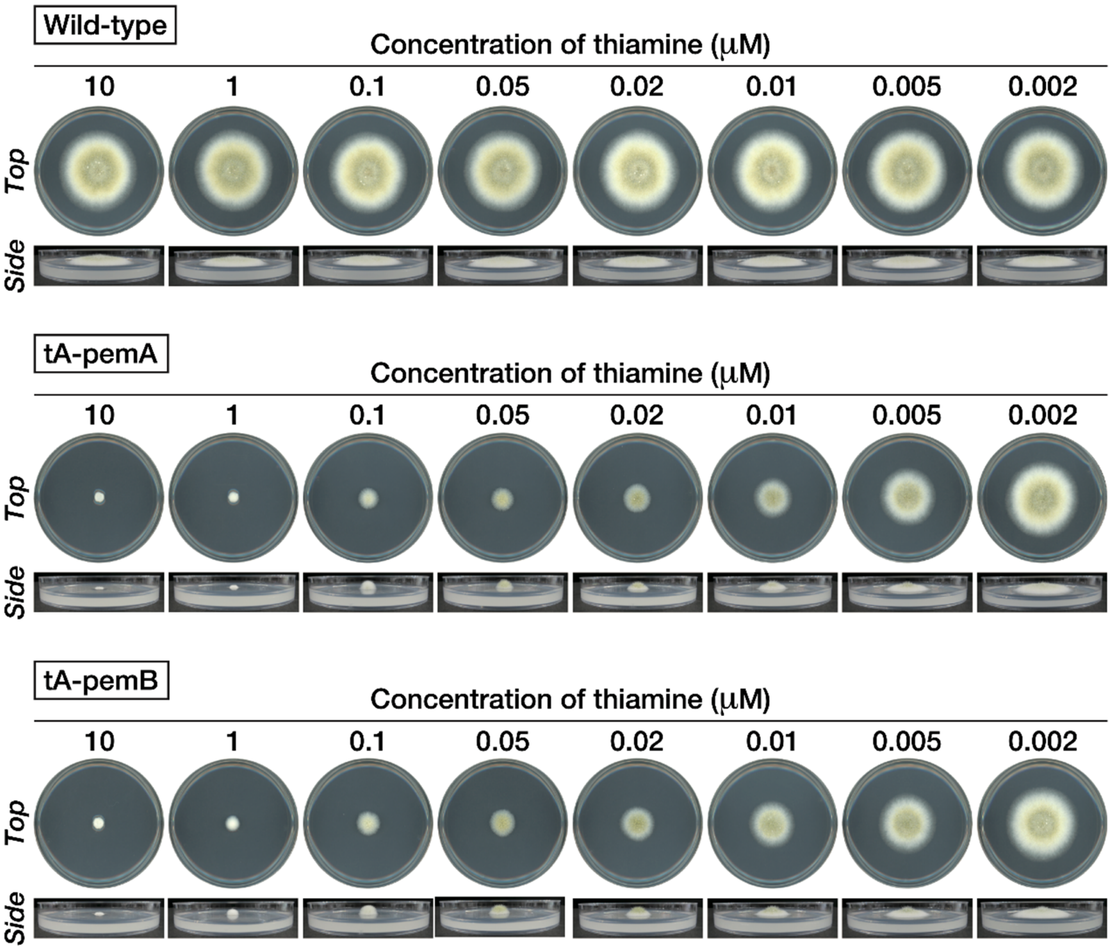

Fig. S8. Growth of the wild-type, tA-pemA, or tA-pemB strain at different concentrations of thiamine.

The conidia of the wild-type, tA-pemA, or tA-pemB were inoculated on a CD medium containing thiamine at different concentrations and incubated at 30°C for 96 h. Top and side views of the colonies that were grown on each concentration of thiamine. The wild-type and tA-pemA colonies at 0.01, 0.1, and 1 μM thiamine were also shown in Fig. 5E.

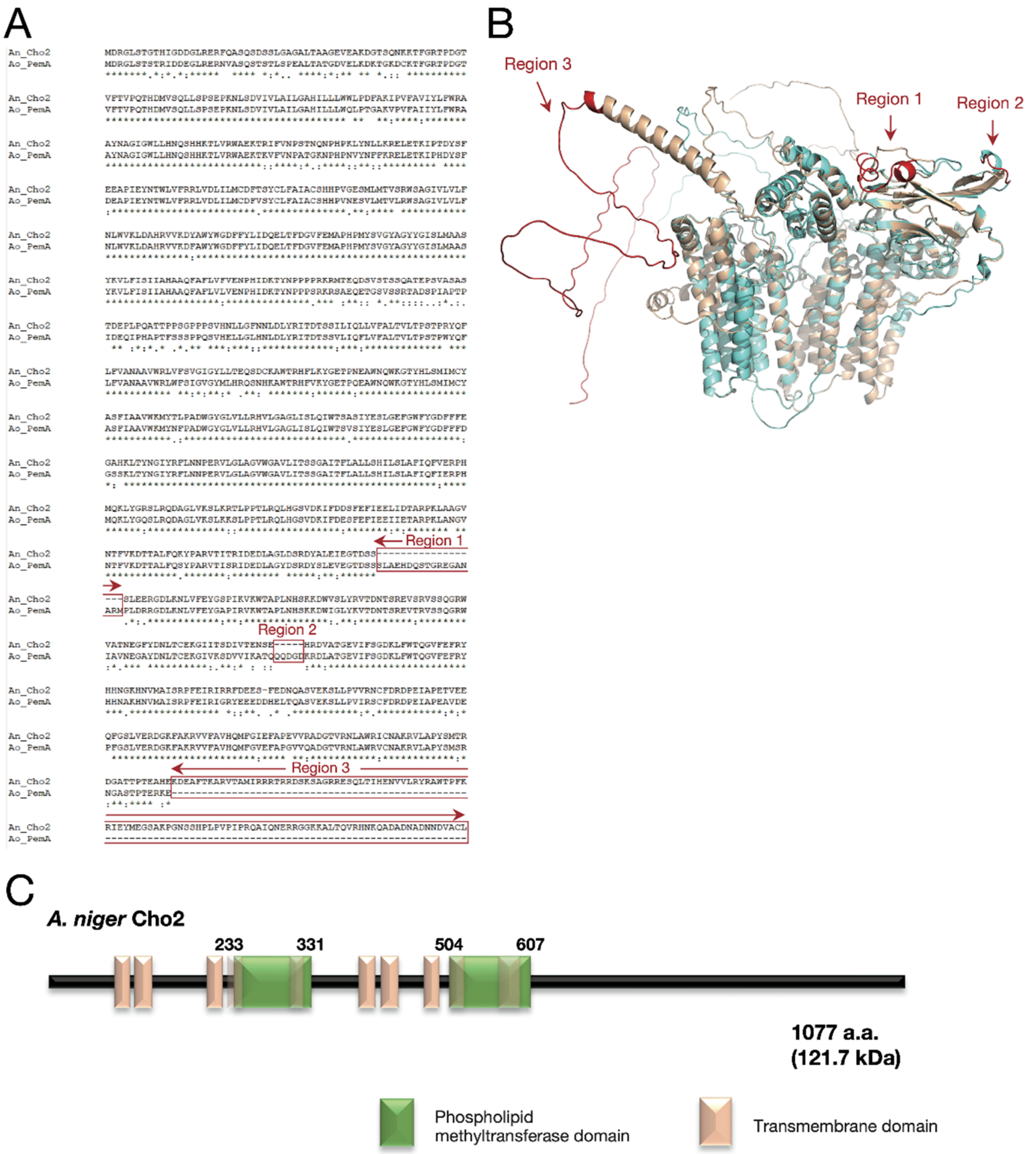

Fig. S9. Comparison of amino acid sequences and putative structures of *A. oryzae* PemA and *A. niger* Cho2.

(A) Amino acid sequence alignments of *A. oryzae* PemA and *A. niger* Cho2. Regions 1 and 2 indicate the amino acid sequences do not present in Cho2. Region 3 indicates the amino acid sequence do not present in PemA. (B) The predicted structures of *A. oryzae* PemA (*cyan*) and *A. niger* Cho2 (*brown*) by AlphaFold2. Regions 1–3 correspond to the regions shown in (A). (C) The deduced domain organizations of *A. niger* Cho2 by InterPro.

Table S1. Genes involved in the Kennedy pathway in *S. cerevisiae* and *A. oryzae*

S. cerevisiae			*A. oryzae*				
Protein name	Alias	SGD ID	Protein name	Accession number	BLASTP E-value	Gene name	Locus tag
Eki1		S000002554	EkiA			*ekiA*	AO090138000211
Ect1	Muq1	S000003239	EctA	XP_001821490.1	2e-53	*ectA*	AO090023001003
Ept1		S000001165	EptA			*eptA*	AO090020000724
Cki1		S000004123	CkiA	XP_023089225.1	2e-71	*ckiA*	AO090005001098
Pct1	Cct1, Bsr2	S000003434	CctA	XP_001818501.1	7e-101	*cctA*	AO090005001594
Cpt1		S000005074	CptA	XP_001817688.3	3e-40	*cptA*	AO090005000648

Table S2. Strains used in this study

Strain	Genotype	Source
RIB40	Wild-type	NRIB*
NSPlD1	*niaD*$^-$ *sC*$^-$ *adeA*$^-$ Δ*argB*::*adeA*	
	Δ*ligD*::*argB* Δ*pyrG*::*adeA*	(49)
R40Δku5-2	Δ*ku70*	(47)
R40Δku5-2ΔpyrG	Δ*ku70* Δ*pyrG*	Katayama, unpublished
Δ*pemA*	Δ*ku70* Δ*pyrG* Δ*pemA*::*pyrG*	This study
Δ*pemB*	Δ*ku70* Δ*pyrG* Δ*pemB*::*pyrG*	This study
tA-pemA	Δ*ku70* Δ*pyrG* *pemA*::*pyrG*-*thiA*p-*pemA*	This study
tA-pemB	Δ*ku70* Δ*pyrG* *pemB*::*pyrG*-*thiA*p-*pemB*	This study

*The National Research Institute of Brewing: Higashihiroshima, Hiroshima, Japan

Table S3. Plasmids used in this study

Plasmid	Description	Source
pUC18	A cloning vector	TaKaRa
pBluescript II SK (+)	A cloning vector	Stratagene
p18-pG	pUC18 carrying *pyrG*	This study
p18-ΔpemA	pUC18 carrying a fragment containing	
	an upstream and a downstream region of *pemA*	This study
p18-ΔpemA-pG	A plasmid for deletion of *pemA*	This study
p18-ΔpemB	pUC18 carrying a fragment containing	
	an upstream and a downstream region of *pemB*	This study
p18-ΔpemB-pG	A plasmid for deletion of *pemB*	This study
p18-pG-tA	pUC18 carrying *pyrG-thiA*p fragment	This study
pBS-pG-tA-pemA	A plasmid to replace *pemA* promoter with *thiA*p	This study
pBS-pG-tA-pemB	A plasmid to replace *pemB* promoter with *thiA*p	This study

Table S4. Primers used in this study

Name	Sequence (5´→3´)
**For construction of plasmids**	
TSAP1	TCGACTCTAGAGGATCCCCAGGTTATATGCTGCCACTGGTGG
TSAP2	GAATTCGAGCTCGGTACCCAGATTGTACGAACAGATGGCCCG
TSAP15	AGATTGTACGAACAGATGGC
TSAP25	CCATCTGTTCGTACAATCTTTCGGTAAATACACTATCACACA
TSAP34	GAATTCGAGCTCGGTACCCGTTTCAAGTTGCAATGACTATCA
TSAP103	TATCGAATTCCTGCAGCCCTTGTGCAAAGAGCATATCGGG
TSAP104	GGGGATCCTCTAGAGTCGAGATTGAGACTCAGCAGAGCAA
TSAP106	AGTCATTGCAACTTGAAACATGGATCGTGGACTTTCAACA
TSAP108	TATCGAATTCCTGCAGCCCACTTCAGTCACGAATGCTGGG
TSAP109	GGGGATCCTCTAGAGTCGATGTGTAGAAAATTAACCCAGG
TSAP111	AGTCATTGCAACTTGAAACATGTCTACTCTTTCGGATTTC
TSAP112	TAGAACTAGTGGATCCCCCAATCCTTTAAGCGTGGGACCC
TSAP124	GTTTCAAGTTGCAATGACTATCA
TSAP126	TAGAACTAGTGGATCCCCCATACCGTAATAGCCAGCGTAGCC
TSAP132	AGGTTATATGCTGCCACTGG
TSAP184	TCGACTCTAGAGGATCCCCTTGCCATTTCGTCTATAGTGTTC
TSAP185	GATTGAGACTCAGCAGAGCAAAATG
TSAP187	GCTCTGCTGAGTCTCAATCGGTGTAAAAGTCACTTGGTGCTT
TSAP188	GAATTCGAGCTCGGTACCCAATCCATTCAATGATCTCCTTTG
TSAP189	TCGACTCTAGAGGATCCCCAAGAAGGCGACTGAAGCAAGCAG
TSAP190	TGTGTAGAAAATTAACCCAGGCTCAGG
TSAP192	TGGGTTAATTTTCTACACAAAGTGCTATGGTACCGCGGCCAG
TSAP193	GAATTCGAGCTCGGTACCCGGAGGAGACTGTCAGGGGATTCG
TSAP237	CAGTGGCAGCATATAACCTGATTGAGACTCAGCAGAGCAAAA
TSAP238	CCATCTGTTCGTACAATCTGGTGTAAAAGTCACTTGGTGCTT
TSAP239	CAGTGGCAGCATATAACCTTGTGTAGAAAATTAACCCAGGCT
TSAP240	CCATCTGTTCGTACAATCTAAGTGCTATGGTACCGCGGCCAG
CGUP1_UF1	TCGACTCTAGAGGATCCCC
CGUP2_UR1	GAATTCGAGCTCGGTACCC
CGUP3_UF2	TATCGAATTCCTGCAGCCC
CGUP4_UR2	TAGAACTAGTGGATCCCCC
**For 5′-RACE**	
pemA_GSP_R	ACCCAGTACACGCTCAGGGTTGTTC
pemA_NGSP_R	AATTCGAGCTCGGTACCCACCAGCCAAATTCACCAAGAGACTC
pemB_GSP_R	ACGGGGGCATCCATGAGAATACCGA
pemB_NGSP_R	AATTCGAGCTCGGTACCCAACGGATCCCACTGCGAACAAAACG

For RT-qPCR	
TSAP204	GTGACTGGAGACGAGGAGACTCATAAC
TSAP205	GTCCACCCATTTACAGTGACGAACAC
TSAP208	GCACGTAGACTGGGAAAGGAACTCTAC
TSAP209	CTTCAACAGCAGATACACGCTCAGTC
TSAP212	GGTGTTGCACTCTACAAGGGCAAAG
TSAP213	TAGATCTCAGCAGTGAAAGGGTCCTC
TSAP230	AGGACTCCACAATCTGGACCTGTATC
TSAP231	GGTGTAGAGGGTGTCAACACTGTGAG
TSAP702	CCACCTGGGAGACCTATCATACACAT
TSAP703	AGACCATATCTCAGGCCCATACCAG
TSAP705	GAGATACTGCGTATCACTCACACGCTAC
TSAP706	CGCTCAGTCGAACTACTCCTACATCAC
TSAP708	CGTCTCAGAAGCAGATCATCGATCGAC
TSAP709	GCCCATATACCCCAGTATAATCCGGGA
TSAP712	GAGAACTAAGCAAAGTGGTCCTCTGGG
TSAP713	GATCCCCAAAGAGTCACAACAGTAGCC
QP037	AACTCCTTTGTCAATGACATCTTCG
QP038	GTCTGAATCTCCCTTGACGAGATAG
